# Silver nanoparticle enhanced metal-organic matrix with interface-engineering for efficient photocatalytic hydrogen evolution

**DOI:** 10.1038/s41467-023-35981-8

**Published:** 2023-02-01

**Authors:** Yannan Liu, Cheng-Hao Liu, Tushar Debnath, Yong Wang, Darius Pohl, Lucas V. Besteiro, Debora Motta Meira, Shengyun Huang, Fan Yang, Bernd Rellinghaus, Mohamed Chaker, Dmytro F. Perepichka, Dongling Ma

**Affiliations:** 1 Énergie Matériaux et Télécommunications, Institut National de la Recherche Scientifque (INRS) 1650 Boul. Lionel-Boulet, Varennes, QC J3X 1P7 Canada; 2 Department of Chemistry, McGill University, 801 Sherbrooke Street West, Montreal, QC H3A 0B8 Canada; 3 Chair for Photonics and Optoelectronics Nano-Institute Munich Department of Physics, Ludwig-Maximilians-University, Königinstr. 10, 80539 München, Germany; 4 Present Address: Dresden Center for Nanoanalysis (DCN), 01062 Dresden, Germany; 5 CINBIO, Universidade de Vigo, 36310 Vigo, Spain; 6 CLS@APS sector 20, Advanced Photon Source, Argonne National Laboratory, 60439 Lemont, IL USA; 7 Canadian Light Source Inc., Saskatoon, SK S7N 2V3 Canada; 8 Department of Materials Science and Engineering, Stanford University, Stanford, CA 94305 USA; 9 Present Address: Center for Advancing Electronics Dresden (Cfaed), Technische Universität Dresden, 01062 Dresden, Germany

**Keywords:** Photocatalysis, Artificial photosynthesis, Nanoparticles

## Abstract

Integrating plasmonic nanoparticles into the photoactive metal-organic matrix is highly desirable due to the plasmonic near field enhancement, complementary light absorption, and accelerated separation of photogenerated charge carriers at the junction interface. The construction of a well-defined, intimate interface is vital for efficient charge carrier separation, however, it remains a challenge in synthesis. Here we synthesize a junction bearing intimate interface, composed of plasmonic Ag nanoparticles and matrix with silver node via a facile one-step approach. The plasmonic effect of Ag nanoparticles on the matrix is visualized through electron energy loss mapping. Moreover, charge carrier transfer from the plasmonic nanoparticles to the matrix is verified through ultrafast transient absorption spectroscopy and in-situ photoelectron spectroscopy. The system delivers highly efficient visible-light photocatalytic H_2_ generation, surpassing most reported metal-organic framework-based photocatalytic systems. This work sheds light on effective electronic and energy bridging between plasmonic nanoparticles and organic semiconductors.

## Abstract (web summary)

The integration of plasmonic structures with photoactive matrices offers a promising means to enhance solar-to-fuel conversion. Here, the authors bridge plasmonic nanoparticles and metal-organic matrix through interface-engineering to boost photocatalytic hydrogen evolution.

## Introduction

Converting solar energy to clean H_2_ fuel by photocatalysis is a promising strategy for sustainable human development^1–3^. The grand challenge in the photocatalytic area is the relatively low efficiency, suffering from the narrow light absorption and severe photogenerated hole and electron recombination^4–6^. Thus achieving extensive visible-light absorption and efficient solar-to-chemical energy conversion is a sought-after goal. Plasmonic nanoparticles (NPs) featuring surface plasmon resonance (SPR) are ideal photocatalytic candidates to extend the photo-responsive range and increase exciton generation of semiconductors by enhanced electromagnetic near fields^7–9^, which has attracted broad interest. Integrating plasmonic NPs into semiconductors has two prominent advantages: (i) the formation of a Schottky junction, which contributes to charge separation and transfer; (ii) localized surface plasmonic resonance (LSPR), which is beneficial to the extended photon absorption range and the excitation of active charge carriers.

Compared with most reported inorganic semiconductors, metal-organic matrix (MOM, such as metal-organic frameworks (MOFs)) semiconductors, formed by coordination-driven self-assembly between metal or metal cluster centers and photoactive organic ligands, shows great potential in photocatalysis due to excellent structural designability and the power of regular porous structure^10,11^. Therefore, a hybrid made of plasmonic NPs and photoactive MOM would be of significant interest for photocatalytic applications^12,13^. However, one of the biggest challenges in constructing such a junction is preparing a well-defined and surfactant-free, ‘intimate’ interface: surfactants at the interface can act as a barrier and result in poor charge transport between the components, thus suppressing the photocatalytic activity^14^. Realizing a well-defined, barrier-free interface is thus essential for understanding the fundamental interplay between the two constituents and for the realization of practical photocatalytic applications. For instance, Ag (I) cluster-based nodes could allow an effective ligand-to-metal charge transfer (LMCT) or metal/cluster-to-ligand charge transfer (MLCT/CLCT), which would help the photocatalysis^15^. However, very few Ag ion cluster-containing MOMs have been reported because they tend to suffer from poor stability due to the weak Ag-ligand coordination bond^6^.

**Fig. 1:**
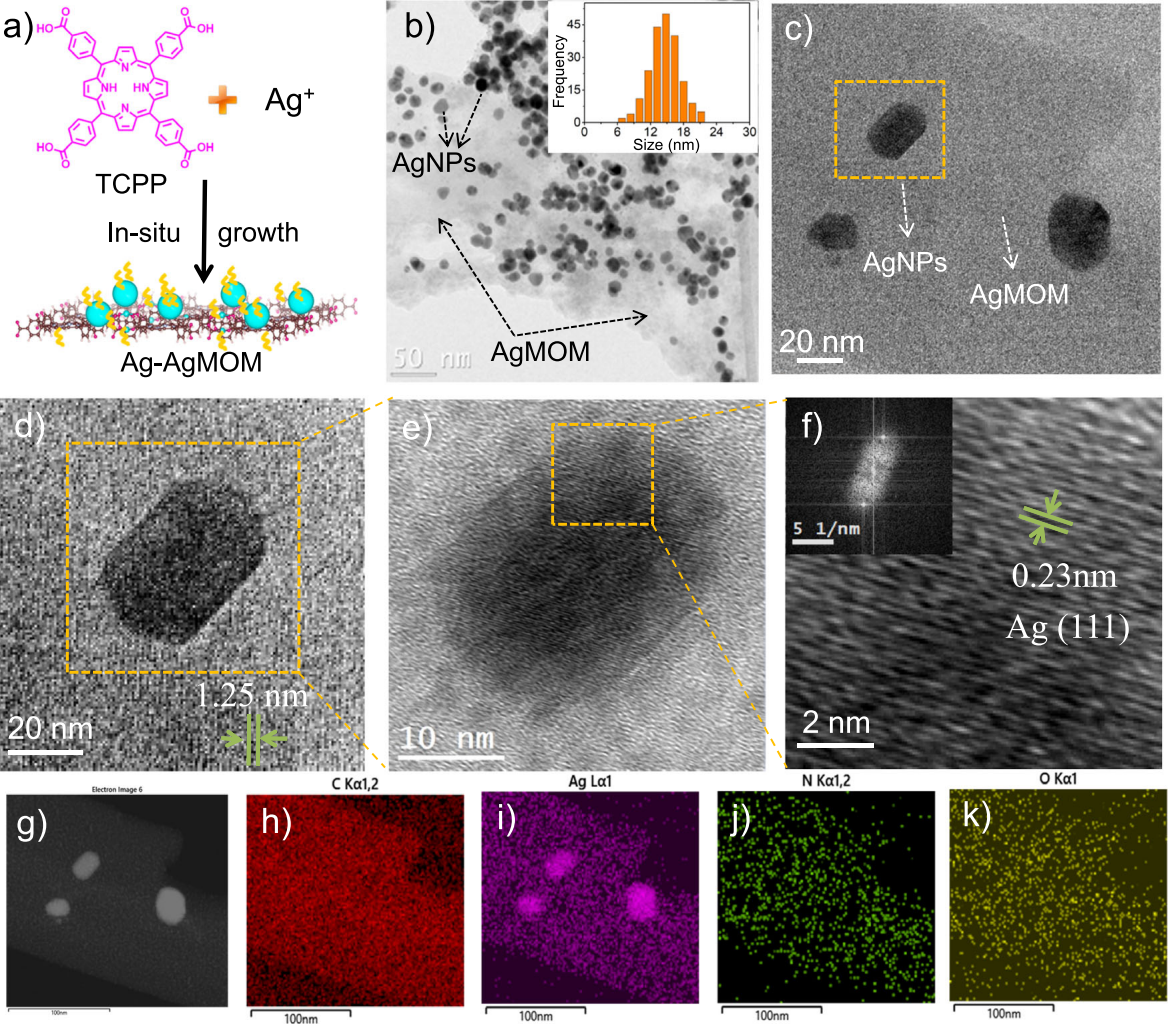
Morphology characterizations of Ag–AgMOM. **a** Schematic diagram of the one-step synthesis of Ag–AgMOM using in-situ solvothermal method. **b** Typical low magnified TEM image of Ag–AgMOM with reaction time 28 h. Inset is the statistical size distribution of Ag nanoparticles (AgNPs). **c**, **d** Enlarged TEM images of Ag–AgMOM; the lattice of AgMOM was observed. **e**, **f** HR-TEM images and crystal lattice of a single Ag nanoparticle (AgNP) on AgMOM in (**d**) (inset is the FFT image of the AgNP). **g** High-angle annular dark-field (HAADF) image of Ag–AgMOM. **h**–**k** Mapping of carbon (C), silver (Ag), nitrogen (N) and oxygen (O) elements in Ag–AgMOM, respectively. Source data are provided as a Source Data file.

In this work, we develop a facile one-step approach to construct a hybrid structure of plasmonic AgNPs and Ag-porphyrin MOM, denoted as Ag–AgMOMs. The in-situ growth strategy enables the AgNPs and AgMOM to share the Ag atoms at the interface, which appears essential for an intimate interface between the two compounds (Fig. 1a). A “clean” metal-MOM interface with few surfactants is demonstrated by surface-enhanced Raman spectroscopy (SERS) and related control experiments. The lack of surfactants (polyvinylpyrrolidone, PVP) at the inner NP/MOM interface allows the efficient interfacial charge transfer, while PVP on the outer surface of Ag–AgMOMs imparts the excellent dispersibility and stability of the latter in aqueous solution. Electron energy loss spectroscopy (EELS) spectra and mapping offer the observation of the plasmonic effect of AgNPs on the AgMOM. The plasmonic AgNPs significantly enhance the photocatalytic hydrogen evolution of the AgMOM, reaching high values of 1025 and 3153 μmol h^−1^ g^−1^ under visible-light and full-spectrum light irradiation, respectively. These values are comparable with those of the state-of-the-art metal-organic-based photocatalytic systems^5,16^. The underlying charge transfer from the plasmonic AgNPs to AgMOM, exciton-plasmon coupling and associated dynamics under light irradiation are further revealed by in-situ X-ray photoelectron spectroscopy (XPS) and ultrafast transient absorption (TA) spectroscopy. This work may guide the design of plasmonic nanoparticles coupled with organic semiconductors for effective electronic and energy communication.

## Results

### Preparation and characterization of Ag–AgMOMs

The target sample of Ag–AgMOM (28 h) (abbreviated as Ag–AgMOM) was synthesized through a one-step solvothermal method. Silver trifluoroacetate and tetrakis(4-carboxyphenyl)porphyrin (TCPP) act as the silver source and photoactive organic ligand, respectively. The morphology of as-prepared Ag–AgMOM was characterized by transmission electron microscopy (TEM) and atomic force microscopy (AFM). As shown in Figs. 1b and 1c, a large number of (quasi-)spherical NPs were present in the matrix a after a reaction time of 28 h with a high yield (ca. 89 %) and a precursor ratio of m_Ag+_/m_TCPP_ = 1:1, and no free NPs (not anchored on the matrix) were observed on the TEM grid. The average diameter of NPs was 14.7 ± 3.5 nm, based on the statistical analyses of 200 NPs in the TEM images (inset in Fig. 1b). The lattice fringes of AgMOM around AgNPs (Fig. 1d, Supplementary Figs. 1 and 2b, c) with a spacing of ca. 1.25 nm were observed. Moreover, the high-resolution TEM (HR-TEM) image of NPs revealed their lattice fringes with a spacing of 0.23 nm (Figs. 1e, 1f and Supplementary Fig. 1c, d), which is attributed to the (111) crystal planes of AgNPs^17^. The (111) crystal plane is preferrentially exposed   due to its lowest surface energy (ca. 0.76 J/m^2^) compared with other crystal planes in cubic-structured Ag crystals^18^. Moreover, the fast Fourier-transform (FFT) pattern (inset in Fig. 1f) of the NP presents the characteristic diffraction pattern of the face-centered cubic (FCC) crystal structure of silver crystal (Supplementary Fig. 3). High-angle annular dark-field (HAADF) imaging of Ag–AgMOM (Fig. 1g) and element mapping of carbon (C, Fig. 1h), silver (Ag, Fig. 1i), nitrogen (N, Fig. 1j) and oxygen (O, Fig. 1k) was performed by a scanning transmission electron microscope (STEM). The MOM (in the area without obvious AgNPs, Fig. 1i) displayed strong signals of C, N, O, and Ag elements, while the area occupied by NPs presented a much stronger Ag signal, further confirming that the samples are composed of AgMOM containing Ag element and AgNPs. The elements of Ag–AgMOM were also determined by the energy dispersive X-ray spectrum (Supplementary Fig. 4). The overall concentration of Ag in the Ag–AgMOM was determined to be 58.9 ± 3.5 wt% via the neutron activation analysis (NAA), indicating a high content of Ag in the composite sample.

**Fig. 2:**
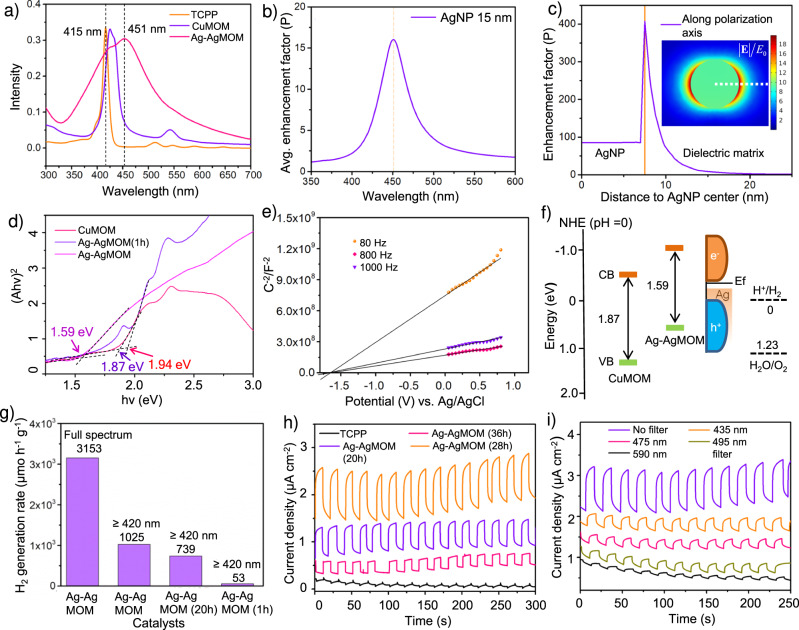
Optical and electronic properties and photocatalytic performance of Ag–AgMOM. **a** Ultraviolet-visible absorption spectra of Ag–AgMOM, tetrakis(4-carboxyphenyl)porphyrin (TCPP) and CuMOM in ethanol solution. **b** Simulation spectrum of the average enhancement factor in a shell surrounding the AgNP of 15 nm in diameter, embedded in MOF. **c** Enhancement factor along the polarization axis, under a planewave excitation with a wavelength of 451.3 nm. Inset: map of the normalized electric field along a cross section of the Ag nanoparticle (AgNP) at the same wavelength. **d** The Tauc plots of CuMOM, Ag–AgMOM and Ag–AgMOM(1 h). **e** Mott–Schottky plots of Ag–AgMOM under different frequencies. **f** Energy levels of CuMOM, Ag–AgMOM and AgNPs (NHE, pH = 0). **g** Hydrogen generation rate under 300 W irradiation in the presence of Ag–AgMOM synthesized at reaction time of 1 h, 20 h and 28 h with or without a 420 nm long-pass filter, respectively. **h** Transient photocurrent density spectra of TCPP and Ag–AgMOM under 150 W solar simulation irradiation at a bias of 0.2 V vs. Ag/AgCl. **i** Transient photocurrent density spectra of Ag–AgMOM (28 h) measured using different long-pass filters of 435 nm, 475 nm, 495 nm and 590 nm at a bias of 0.2 V vs. Ag/AgCl. Source data are provided as a Source Data file.

The topography image (Supplementary Fig. 5a) and the corresponding height measurements (Supplementary Fig. 5b) of Ag–AgMOM samples were taken using atomic force microscopy (AFM). Samples were drop-casted onto the V1 AFM mica disc, the roughness of which was measured to be smaller than 1 nm (profile 4 in Supplementary Fig. 5a, b). The average thickness of AgMOM (profiles 1, 2, 3 in Supplementary Fig. 5a, b) was measured to be ca. 5 nm, reflecting the thin feature of AgMOM. We speculated that the formation of such ultrathin AgMOM is due to the synergistic effect of PVP attachment and in-situ growth of AgNPs on the (001) surface of AgMOM, which inhibits the Z-axis growth of AgMOM^19^.

Further characterizations of the chemical structure were performed by Fourier-transform infrared spectroscopy (FTIR). As displayed in Supplementary Fig. 6, FTIR of TCPP monomer showed C=O stretching from the free carboxylic (COOH) group at 1695 cm^−1^ is significantly weakened while the carboxylate peak at 1671 cm^−1^ is markedly increased. Furthermore, FTIR of TCPP showed a broad H-bonding peak around 3000 cm^−1^, whereas this peak essentially disappeared in Ag–AgMOM, indicative of the absence of free OH groups. We thus believe the carboxylic groups in TCPP are coordinated with Ag ions^20^. It should be noted that the as-prepared Ag–AgMOM sample dispersed well in water solution (Supplementary Fig. 7) in virtue of the PVP coverage on the surface. The high hydrophilicity is supported by a small water contact angle of 32° for the Ag–AgMOM film (Supplementary Fig. 8). Of note, such a small contact angle is favorable for photosplitting of water^1^.

### Controllable synthesis and growth mechanism of Ag–AgMOMs

It is well known that the SPR characteristics of AgNPs highly depend on their size and shape. In this work, the size of AgNPs in the Ag–AgMOM systems was tuned via the reaction time, feeding ratio and regulating agent during the one-step synthesis. As displayed in Supplementary Figs. 9–11, the average diameter of AgNPs could be reduced from 14.7 nm to 3.2 nm, 7.9 nm and 12.5 nm when the reaction time was decreased from 28 h to 20 h, 4 h and 1 h, respectively, when maintaining all other synthetic parameters. When the reaction time was extended to 36 h, the average diameter of AgNPs reached up to ~19.3 nm (Supplementary Fig. 12). The use of stoichiometric  excess of Ag ions (weight ratio of Ag salt/TCPP from 1:1 to 2:1) led to a dramatically increased coverage density of NPs with a slightly larger average diameter of 15.2 nm (Supplementary Fig. 13). In contrast, decreasing the ratio of Ag salt/TCPP to 1:4 resulted in a lower coverage and a smaller size (8.5 nm) of AgNPs (Supplementary Fig. 14). A moderate coverage density of AgNPs with an average diameter of 7.2 nm was obtained using *m*_Ag+_/*m*_TCPP_ = 1:2 at the 4 h reaction (Supplementary Fig. 15). The effect of conditions on AgNP size  is presented in Supplementary Fig. 16. In all the cases, no observed free AgNPs suggests that AgNP nucleation happens in the AgMOM rather than in the homogeneous reaction solution (mixture of dimethylformamide (DMF) and ethanol).

To gain more tunability on the NP’s size and heterostructure’s morphology, we further introduced a modulator (trifluoroacetic acid, TFA). As shown in Supplementary Fig. 17, a fiber-like Ag–AgMOM structure with ultrasmall AgNPs of 2.8 nm in diameter was formed by adding an additional 100 μL TFA into the reaction system (*m*_Ag+_/*m*_TCPP_ = 1:1, 28 h), manifesting that TFA suppresses the growth of AgNPs and influences the formation of Ag–AgMOM. These effects were also evidenced by forming smaller AgNPs of 1.7 nm (Supplementary Fig. 18 and S19) as increasing the feeding amount of triethanolamine (TEOA) to 200 μL. The conditions are summarized in Supplementary Fig. 20.

As a control, free Ag nanocubes (without MOM) were synthesized under the same reaction condition as that for synthesizing the Ag–AgMOM (28 h) sample except for the absence of the TCPP ligand (Supplementary Fig. 21). The different shape of Ag nanocubes compared with the AgNPs in the Ag–AgMOM indicates the restricted growth of AgNPs during the preparation of Ag–AgMOM. Smaller AgNPs (~3 nm) were obtained in Cu-porphyrin matrix (named as Ag-CuMOM) by introducing Cu^2+^ ions into the reaction system (*m*_Cu2+_/*m*_Ag+_/*m*_TCPP_ = 1:1:1, 28 h) (Supplementary Fig. 22). The stronger coordination bonding of Cu_2_(COO)_4_ compared to Ag_2_(COO)_4_ possibly interrupted the formation of Cu-porphyrin MOM rather than Ag-porphyrin MOM in the above condition, as evidenced by EDS analysis of Ag-CuMOM in the varied areas (Supplementary Fig. 23). Like Ag–AgMOM, Ag–CuMOM was stabilized in water by PVP surfactant and displayed a strong Tyndall effect (Supplementary Fig. 24).

Combining all the results, we propose the following mechanism of Ag–AgMOM formation. Firstly, the dissolved Ag ions undergo the metal-organic coordination with the TCPP ligand, forming AgMOM. Then the Ag ion nodes in the AgMOM act as nucleation sites to induce the heterogeneous growth of AgNPs. In turn, the AgNPs stabilized the AgMOM by presumably sharing the Ag atoms at the interface. As a result, a metal-MOM hybrid composed of AgNPs and AgMOM with a rather intimate interface is successfully prepared.

### Optical and electronic properties of Ag–AgMOM

Photoabsorptivity and energy level alignment of materials play key roles in photocatalytic performances. The optical features of Ag–AgMOM were investigated using ultraviolet-visible (UV–Vis) absorption spectroscopy. As displayed in Fig. 2a, a broad peak at ~451 nm with a shoulder at ~430 nm was detected in the UV-Vis absorption spectrum of Ag–AgMOM. This absorption at ~430 nm corresponds to the B band absorption of porphyrins. The red-shift (compared with the peak of pure TCPP at 415 nm) and significant broadening of this peak suggest the stacking of porphyrins after the formation of MOM. Such stacking could provide charge carrier transport channels, which are essential for charge extraction and for suppressing the detrimental charge recombination^21^. The electromagnetic response of AgNPs was computed using full-wave electrodynamic simulations. Fig. 2b shows the spectral response of a single particle, simulated as the volume average of the enhancement factor ${P}={|{E}|}^{2}/{{E}}_{0}^{2}$ over a 10 nm shell surrounding the NP, through which the magnitude of the electric field is referenced to the amplitude of the external driving field (*E*_0_) in the near-field region of the AgNP. The simulated spectrum of the average enhancement factor in a shell surrounding the AgNP with a peak position at 450 nm suggested the broad peak at 451.3 nm in the Ag–AgMOM system can mainly be ascribed to the SPR band of AgNPs (Fig. 2b)^7,22^. As shown in Fig. 2c, the independent magnitude *r* is the radial distance to the center of the AgNP, and the surface of the sphere is marked by a yellow line in the plot, separating the “AgNP” and “Dielectric matrix” regions. The near-field enhancement at the resonant wavelength of this AgNP is significant due to the surface charges dynamically accumulating at the AgNP surfaces, but it decays rapidly with the distance to its surface (Fig. 2c)^23^. A short distance between the AgNP and semiconductor is thus required in order to fully benefit from the near-field effect.

For a better understanding of the SPR effects on photocatalysis, it is desirable to study the plain MOM with Ag ion coordination but without any AgNP as a control sample. However, we found that it was challenging to synthesize the plain AgMOM without AgNPs because of the relative instability of Ag ion coordinated TCPP itself in the absence of AgNPs. Therefore, we introduced a MOM with Cu^2+^ node (CuMOM) as a control sample to better interpret the electronic structure of the Ag–AgMOM. The UV–Vis spectra of the Ag–AgMOM film displayed an absorption edge at 856 nm and an extended light-harvesting range with respect to the AgNP-free CuMOM (Supplementary Fig. 25), in line with the solution absorption spectra. The optical band gap (Eg) of CuMOM was determined to be 1.87 eV from the Tauc plot (Fig. 2d), consistent with the literature reported values^24^. On the other hand, as displayed in Fig. 2d, Supplementary Fig. 25 and Supplementary Table 1, the optical bandgap of Ag–AgMOM was estimated to be 1.59 eV, smaller than that of Ag–AgMOM (1 h) (1.94 eV), since the latter sample shows quite low LSPR due to the ultrasmall size of AgNPs. Mott-Schottky plots of Ag–AgMOM under different frequencies were employed to determine their conduction band minimum (CBM) (Fig. 2e and Supplementary Fig. 26). Higher CBM (−1.01 V vs. NHE) of Ag–AgMOM than 0 V vs. NHE of water reduction potential implies the feasiblity of Ag–AgMOM for photocatalytic H_2_ generation. Overall energy alignments of CuMOM, Ag–AgMOM and AgNPs (NHE, pH = 0) are presented in Fig. 2f.

### Photocatalytic performance of Ag–AgMOM

Photocatalytic hydrogen evolution tests of Ag–AgMOM were performed in aqueous solutions containing TEOA as a hole scavenger under light irradiation (300 W, Xe lamp) at a constant temperature of 15 °C. The optimized H_2_ production rates for the sample Ag–AgMOM (28 h) reached up to 1025 μmol h^−1^ g^−1^ and 3153 μmol h^−1^ g^−1^ under visible light (with a 420 nm long-pass filter) and full-spectrum light (without any filter) irradiation of a 300 W Xe lamp, respectively. As expected, no H_2_ was detected in the dark for the same sample. As a control, only very low H_2_ generation rate ~ 4 μmol h^−1^ g^−1^ was detected from TCPP itself (Supplementary Fig. 27). Considering almost no plasmonic peak was observed in Ag-AgMOM (1 h) from the UV–Vis absorption spectra due to the very small AgNPs loading (Supplementary Fig. 28), Ag–AgMOM (1 h) was also used as a control sample. The visible-light photocatalytic H_2_ generation rate of Ag–AgMOM (1 h) was tested to be only 57 μmol h^−1^ g^−1^. Meanwhile, few H_2_ (15 μmol h^−1^ g^−1^) was detected for pure CuMOM due to severe charge recombination and relatively narrow absorption range; Ag-CuMOM with small AgNPs (<3 nm) (and no detectable plasmonic effect) presented a slightly higher but still low H_2_ generation rate of 73 μmol h^−1^ g^−1^ (Supplementary Fig. 29). We recognize that the small AgNPs in our system may contribute to photocatalytic activity by acting as a co-catalyst. However, the much higher photocatalytic rate (1025 μmol h^−1^ g^−1^) for the Ag–AgMOM (28 h) compared with 57 μmol h^−1^ g^−1^ for the Ag–AgMOM (1 h) and 73 μmol h^−1^ g^−1^ for the Ag-CuMOM, which already had many tiny AgNPs on the surface, highlights the vital role of the plasmonic effect in the optimal sample of the Ag–AgMOM (28 h). The AgNPs in our optimal sample not only increase the light absorption but also promote the exciton generation of AgMOM by the intense electromagnetic near field. It should be noted that it is the interplay of all related factors (such as plasmonic effect, size and percentage) that determines the final photocatalytic performance. Of note, we cannot exclude the possibility that sacrificial agents, besides H_2_O, may also yield H_2_. But since our materials cannot fully split water by themselves, as noticed for most MOFs and MOMs, we have to involve the use of the sacrificial agent. However, we would like to emphasize that herein we focus on comparing photocatalytic performance of plasmon-enabled Ag–AgMOM with plasmonic effect-free pure CuMOM and Ag–AgMOM (1 h) during the photocatalysis process. The observed dramatic increase of photocatalytic efficiency of the plasmonic sample cannot be explained by the sacrificial agent. The obtained H_2_ production rates are relatively high and comparable with those of the state-of-art metal-organic material (including MOFs) based photocatalysts (Supplementary Table 2)^5,16,25–29^.

We then studied the stability of the Ag–AgMOM after going through photocatalytic reactions under illumination by taking a photograph of Ag–AgMOM in solution before and after the photocatalytic reaction for 200 min (Supplementary Figs. 30 and 31). They did not show any significant difference in color and dispersion. As for the crystalline structure, the PXRD patterns of the Ag–AgMOM remained similar, but the crystallinity became slightly lower after the reaction for 200 min (Supplementary Fig. 32). Moreover, the cycling performance of the Ag–AgMOM was carried out, as displayed in Supplementary Fig. 33. It was found that the H_2_ generation rate of the Ag–AgMOM gradually decreased by ~20 % within 3 cycles (200 min/each cycle), which may be due to the reduced crystallinity of the AgMOM substrate during the photocatalytic reaction.

Transient photocurrent density spectra of TCPP and Ag–AgMOM upon 150 W solar light irradiation (1.5 G, 100 mW cm^−2^) are shown in Fig. 2h. As expected, Ag–AgMOM displayed enhanced photocurrent density compared to TCPP due to its extended light absorption and promoted transfer of photoinduced electrons and holes. Moreover, the transient photocurrent densities of Ag–AgMOM were measured with long-pass filters of 435, 475, 495 and 590 nm (Fig. 2i)^30^. Regarding the photocurrent stability, as shown in Supplementary Fig. 34, the photocurrent of the Ag–AgMOM coated onto FTO glasses was maintained constant within 1000 s under 1 sun irradiation at a bias voltage of 0.4 V (vs. Ag/AgCl). Its stability was also assessed at a bias of −0.6 V for 50 min (Supplementary Fig. 35).

### Chemical and crystal structures of Ag–AgMOM

**Fig. 3:**
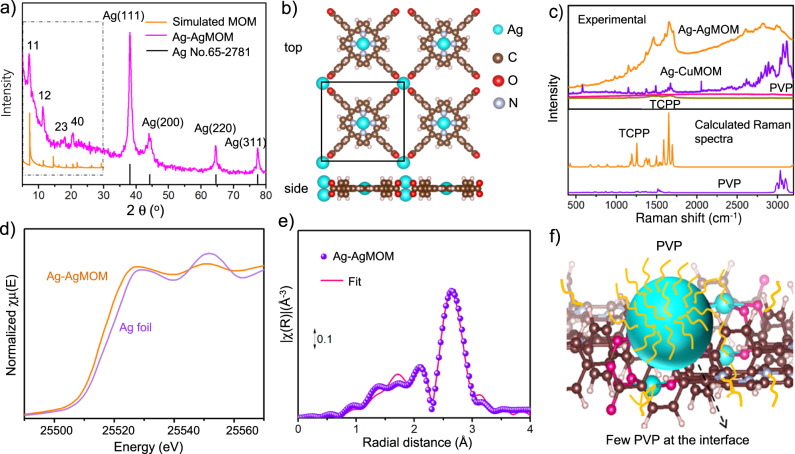
Structural characterizations of Ag–AgMOM. **a** XRD pattern of Ag–AgMOM, Pawley refinement of porphyrin based MOM from 5° to 25° and the standard pattern of Ag (JCPDS No. 65-2871) from 5° to 80°. **b** DFT optimized crystal model of porphyrin based MOM in composites. **c** Raman spectra of Ag–AgMOM, Ag-CuMOM, TCPP and polyvinylpyrrolidone (PVP) taken under the excitation of a 520 nm laser, and DFT calculated Raman spectra of TCPP and PVP (bottom figure). **d** Normalized X-ray absorption near-edge structure (XANES) spectra at Ag K-edge for the Ag–AgMOM sample and Ag foil. **e** Fourier transform of k^2^-weighted extended X-ray absorption fine structure (EXAFS) spectrum (not phase corrected) and best fitting for the Ag–AgMOM sample. **f** Schematic illustration of the model of a AgNP chemically linked with MOM and pristine interface (few PVP at the interface). Source data are provided as a Source Data file.

To elucidate the underlying photocatalytic mechanism, we investigated the structure of the metal-organic matrix and its interface with AgNPs. Figure 3a shows the XRD pattern of AgMOM and the standard pattern of Ag (JCPDS No. 65-2871)^31^. The peaks at 38.5°, 44.2°, 64.4° and 77.4° are ascribed to the (111), (200), (220) and (311) diffractions of FCC Ag crystal, respectively (Supplementary Fig. 1). It should be noted that no apparent XRD peaks of Ag_2_O or AgO were detected, suggesting a lack of oxidation. The other peaks at 2θ = 7.0°, 11.4°, 17.9° and 20.5° are therefore attributed to the Ag–AgMOM, indicating its ordered structure, in line with the TEM observation of AgMOM lattice (Fig. 1d and Supplementary Fig. 1b)^32^.

Unrestricted periodic density-functional theory (DFT) calculations with PBEsol functional and scalar relativistic ZORA correction were subsequently used to optimize a tetragonal model of Ag–AgMOM with a unit cell *a* = *b* = 17.04 Å; *α* = *β* = 90° (Fig. 3b). The simulated diffraction pattern of such a 2D metal-organic network is in good agreement with the experimental XRD (Fig. 3a). As displayed in Supplementary Fig. 36, Pawley refinement of experimental PXRD comparing with the simulated model ranging 2–30° was conducted with acceptable criterion (Rwp = 5.20% and Rp = 3.84%). Orthogonal to the Ag-porphyrin, carboxylate groups in TCPP ligands bridge with a Ag cluster to form the 2D Ag–AgMOM in the D4h symmetry. We note that our simulation employed geometry with 2 Ag ions with 4 -COOH at the metal center. To exclude other possibilities of the AgMOM structure, we checked the model of two silver atoms bonded to two instead of four carboxylates (model 3 in Supplementary Fig. S37), it was found that the simulated PXRD (model 3 in Supplementary Figs. S38 and 39) does not match well with experiment results. We further simulated a model of two silver atoms bonded per three carboxylates, and the resultant simulated PXRD patterns in AA-stacked and in 2D do not match with experimental PXRD pattern of Ag–AgMOM either (Supplementary Fig. 40). Four more different models (e.g., 4 Ag ions with 4 –COOH ligands in various geometries and configurations) for AgMOM were also examined (Supplementary Fig. S37). However, these additional models’ simulated PXRD patterns all show an apparent mismatch to our experimental XRD pattern (Supplementary Figs. 37–40). Moreover, we also excluded the possibility of forming hydrogen-bonded frameworks between the TCPP ligand and Ag only coordinating in the porphyrinic core (Supplementary Fig. 41). These calculations support the proposed structure of AgMOM as a 2D network of TCPP ligands held together by metal-carboxylate interactions. Moreover, it was found the measured PXRD patterns of CuMOM and ZnMOM, containing the same organic ligand of TCPP as the Ag–AgMOM, are pretty similar to that of the Ag–AgMOM in the range of 5-30^o^ (Supplementary Figs. 42 and 43), except for small peak shifts. This is primarily due to slightly varied lattice sizes resulting from different linkage sizes (bond lengths between Ag–O and Cu–O/Zn–O). It should be noted that introducing PVP into the MOM synthesis can increase the water dispersion ability, but decrease the crystallinity of MOMs, regardless of different metal centers (Cu or Zn), explaining our relatively low crystallinity of the Ag–AgMOM.

N_2_ adsorption-desorption measurement was used to examine the porosity and surface area of Ag–AgMOM at 77 K. A rise in the low-pressure range (*P*/*P*_o_ = 0–0.1) in the isotherm plots (Supplementary Fig. 44a) manifests a typical type-I N_2_-sorption isotherm, indicative of the microporous feature of Ag–AgMOM. The Brunauer–Emmett–Teller (BET) specific surface area was determined to be 223 m^2^ g^−1^, which is relatively low due to presence of non-porous Ag nanoparticles and PVP surfactant. Meanwhile, as displayed in Supplementary Fig. 44b, Ag–AgMOM shows angstrom-size pores (ranging from 1.0 to 1.7 nm), derived from nonlocal density-functional theory (NLDFT) calculations of experimental isotherm analyses in Supplementary Fig. 44a, which matches well with the pore size distribution obtained from the simulated AgMOM structure. The BET surface area of the Ag–AgMOM after photocatalysis under full-spectrum light irradiations without a 420 nm filter was further assessed. It was found that the N_2_ adsorption-desorption isotherm of Ag–AgMOM after photocatalysis remained similar to that of the pristine one (Supplementary Fig. 45). However, the specific surface area (151 m^2^ g^−1^) of Ag–AgMOM after light irradiation was a bit lower than that (223 m^2^ g^−1^) of the pristine one, maybe due to slight decreased crystallinity of the Ag–AgMOM (Supplementary Fig. 32) or slight agglomeration after photocatalysis.

The band structure and spin-resolved density of states calculations (Supplementary Fig. 46) of the AgMOM obtained from DFT calculations in the range-separated hybrid functional HSE06 indicates a bandgap of 0.62 eV with VBM at −5.47 eV (−0.97 eV vs. RHE) and CBM at −4.85 eV (−0.35 eV vs. RHE). The larger observed optical bandgap may be attributed to the lack of HOMO-LUMO orbital overlap (oscillator strength for HOMO-LUMO excitation in time-dependent (TD)DFT calculation of the porphyrin-carboxylate fragment is less than 0.001). Indeed, the band structure EK plot of the pristine 2D AgMOM reveals relatively flat bands with large effective masses (>50 m*_h/e_), showing the lack of π-conjugation in the Ag-carboxylate bridges of AgMOM without additional AgNPs.

The Raman spectra of TCPP, PVP and Ag–AgMOM were taken using a 520 nm laser as the excitation source. As shown in Fig. 3c (bottom panel), DFT calculated Raman spectra of TCPP and simplified PVP (repeating unit = 4, Supplementary Fig. 47) are basically in line with their experimental spectra (Fig. 3c, top panel). The enhanced Raman signal of organic TCPP ligands in Ag–AgMOM, with respect to pure TCPP (Fig. 3c, featureless purple line), is attributed to surface-enhanced Raman scattering (SERS) originating from the electromagnetic field enhancement due to the presence of plasmonic AgNPs nearby. Specifically, the significantly enhanced Raman signals (1200~1700 cm^−1^) of porphyrin in AgMOM suggest an intimate interface, with only a few PVP molecules between AgNPs and porphyrin AgMOM^13,33^. Since the near-field decays fast with the distance from the AgNP’s surface (Fig. 2c), the presence of large ligands will significantly decrease the positive contribution of the near field to the Raman signal. To verify our hypothesis of the clean interface, we further synthesized AgNPs of a similar size of ~20 nm and capped with the same PVP ligand (Supplementary Figs. S48 and S49). Subsequently, we loaded them onto the CuMOM as a control sample (Supplementary Figs. S50 and S51). In this case, we could mainly detect the PVP signal but relatively weak TCPP signal due to the inevitable presence of PVP at the interface of AgNPs and CuMOM (Fig. 3c). Indeed, usually it is challenging to remove surfactants from the surface of NPs prepared via traditional synthesis methods, which entail a separate preparation of 2D MOM and NPs, followed by their coupling.

Moreover, the strong Raman band of Ag–AgMOM ranging from 3000 to 3100 cm^−1^, ascribed to PVP (Fig. 3c), likely implies that the rest of AgNPs’ surface (not in contact with porphyrin MOM) is wrapped by the PVP surfactants, which enable the colloidal stability of composites in water. It is worth pointing out that, the considerably increased PVP Raman signals in the composite compared to pure PVP highlight the SERS effect due to AgNPs.

XPS was used to analyze Ag–AgMOM samples synthesized at the reaction time of 1 and 20 h. We found that they both contained Ag ion and metal Ag^0^ (Supplementary Fig. 52). The Ag ion signal at 373.8 eV (or 373.6 eV) and 367.8 eV (or 367.6 eV) is derived from Ag, which is a crucial constituent part of AgMOM and Ag ion species on the surface of AgNPs, whereas the Ag^0^ at 374.2 eV and 368.2 eV is attributed to AgNPs^34^. As shown in Supplementary Table 3, the integrated peak area ratio of Ag^0^ in the Ag–AgMOM (20 h), estimated from the XPS measurements, was markedly increased with respect to the 1 h sample, indicating more or larger AgNPs were formed with reaction time. Interestingly, compared with Ag–AgMOM (1 h), the Ag ion 3*d* XPS peak of Ag–AgMOM (20 h) shifted toward lower binding energy by 0.2 eV (Supplementary Fig. 52a), while the O 1 *s* peak is shifted toward higher binding energy by 0.5 eV (Supplementary Fig. 52b). It suggests that the Ag atoms in AgNPs interact with O atoms in the metal-organic matrix, further supporting the intimate interface between AgNP and AgMOM.

X-ray absorption near-edge structure (XANES) spectra and extended X-ray absorption fine structure (EXAFS) spectrum of Ag–AgMOM were taken to further reveal the linkage. Figure 3d presents the Ag–AgMOM and Ag foil XANES spectra. The damped oscillations are surface effects due to the small size of the particles. The spectrum of Ag–AgMOM is similar to that of Ag foil but slightly shifted to the low energy, indicating the presence of both Ag^0^ and Ag^+^ species^35^. The small coordination numbers (~4) of Ag–AgMOM from the EXAFS spectrum (Fig. 3e and Supplementary Table 4), compared to the reference compounds of Ag foil (12), indicate the formation of small AgNPs in AgMOM. A small Ag–O contribution from the Ag–AgMOM sample suggests the Ag–O bonding existence, in agreement with the XANES results and XPS results.

We thus propose a structural model of Ag–AgMOM with a clean interface (few or no PVP at the interface, shown in Fig. 3f and Supplementary Figs. 53, 54), where our evidence points to a MOM with Ag-porphyrin linkers and -(COO)_4_Ag_2_- nodes. It should be noted that such a –COOAg- motif is generally not very stable due to the weak coordination bonding; to the best of our knowledge, porphyrin MOFs with Ag ion node has rarely been reported. Unfortunately, we failed to attain the pristine AgMOM without the simultaneous formation of AgNPs on the surface despite our considerable attempts. To understand how AgNPs co-exist on the 2D AgMOM, we calculated the binding energy in DFT via energy decomposition analysis (M062X/DZP) of the Ag_3_-AgMOM fragment. We find that there is an additional stabilization of −13.9 kcal/mol resulting from this association, which supports our experimental observation. We thus hypothesize that the in-situ one-step synthesis brings a significant stabilization to the hybrid structure. As a result of the strong interfacial bonding, the AgNPs could not be detached from the porphyrin AgMOM even after strong sonication. Such a strong binding between two ingredients is unusual in nanoparticle-loaded MOM composites but is highly desired for their applications. This junction with the surfactant-free interface enabling charge transfer across the interface is promising for photocatalysis. The Nyquist plot of the Ag–AgMOM hybrid photocatalyst shows a much smaller semicircle radius than that of the TCPP precursor (Supplementary Fig. 55), revealing a lower charge-transfer resistance and faster transport of photogenerated charges of the former, which may also benefit from the pristine interface^36^. The radius of both TCPP and Ag–AgMOM became smaller under light irradiation, suggesting their photo reduction capability.

### Plasmonic and photocatalytic mechanism

**Fig. 4:**
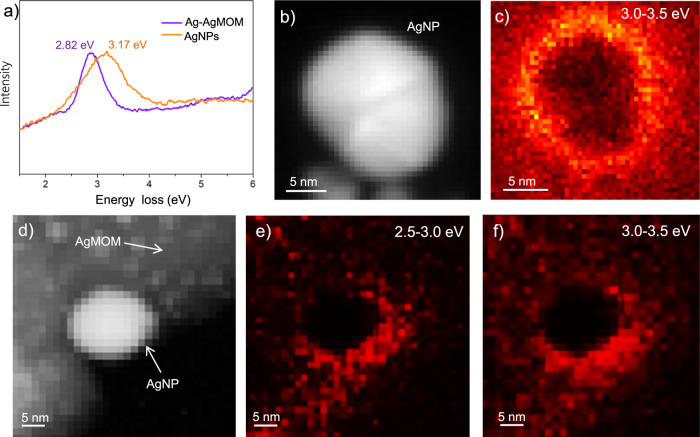
Low-loss EELS and HAADF-STEM characterizations of AgNPs and Ag–AgMOM. **a** Background-subtracted (zero-loss tail removed) EELS spectra. **b** HAADF-STEM image of AgNP and **c** Plasmon mapping of AgNP in the energy range of 3.0~3.5 eV. **d** HAADF-STEM image of Ag–AgMOM and **e**, **f** Plasmon mapping of Ag–AgMOM in two different energy ranges (2.5–3.0 eV and 3.0–3.5 eV). Source data are provided as a Source Data file.

The EELS spectra, after background subtraction, of similarly sized (around 20 nm) and shaped pure AgNPs and those on the Ag–AgMOM are shown in Fig. 4a and Supplementary Fig. 56. It was found that the plasmon resonance peak of AgNPs on the AgMOM displayed lower energy at ca. 2.82 eV compared with that (3.17 eV) of pure AgNPs, suggesting the effect of dielectric environment variation (due to the AgMOM’s presence) on the LSPR of AgNPs^34^. Moreover, both the pure AgNPs (Fig. 4b, c) and the AgNPs on the AgMOM display surface plasmonic excitation (Fig. 4d–f, Supplementary Fig. 56a), which explains the extended light absorption range of Ag–AgMOM. A similar phenomenon becomes more evident in the AgNPs with a larger size (Supplementary Fig. 57). We also studied smaller AgNPs of 6 nm in diameter. In this case, the plasmonic signal is below the limit of detection of the EELS setup, suggesting their much weaker plasmon resonance (if there is any)^37,38^.

**Fig. 5:**
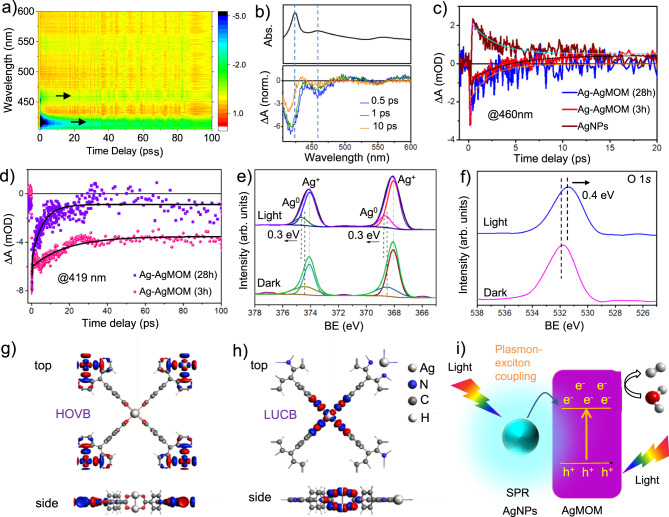
Photocatalytic mechanism studies of Ag–AgMOM photocatalyst. Femtosecond transient absorption spectra in a contour diagram **a** and at selected time delays **b** of Ag–AgMOM (3 h) sample, obtained with a pump wavelength of 400 nm. The steady-state absorption spectrum is also shown at the top in **b** for comparison. **c** Transient time traces at the probe wavelength of 460 nm for AgNPs, Ag–AgMOM (3 h) and Ag–AgMOM (28 h) samples. **d** Transient time traces at the strongest exciton wavelength of 419 nm for both Ag–AgMOM (3 h) and Ag–AgMOM (28 h) samples. High-resolution XPS spectra for Ag 3*d* (**d**), O 1*s* (**e**) and N 1*s* (**f**) of Ag–AgMOM (4 h) with or without 420 nm LED irradiation (BE: binding energy). DFT calculated highest occupied valence band (HOVB) (**g**) and lowest unoccupied conduction band (LUCB) (**h**) topologies of MOM. **i** Proposed visible-driven photocatalytic mechanism of Ag–AgMOM. Source data are provided as a Source Data file.

Femtosecond TA spectroscopy was employed to disclose the interaction between the plasmon from the AgNPs and exciton from the AgMOM^39,40^. Since it is quite challenging to prepare the pure AgMOM without AgNPs, herein Ag–AgMOM (3 h) with a relatively low plasmonic effect and Ag–AgMOM (28 h) with a strong plasmonic effect were investigated and compared. The TA spectra of the Ag–AgMOM (3 h) and Ag–AgMOM (28 h) samples at different time delays after 400 nm pump laser excitation are shown in Fig. 5a, b and Supplementary Fig. 58a. Comparing the TA spectra with corresponding steady-state absorption spectra shown in Figs. 2a and 5b (top), we reasonably infer that the bleaching feature at ~420 nm is mainly derived from the AgMOM related to the Soret band corresponding to the S0–S2 transition excitation of the AgMOM^41,42^. Interestingly, another bleaching signal is observed at ~460 nm, consistent with the absorption spectra of both the Ag–AgMOM (3 h) and Ag–AgMOM (28 h) samples. Such a peak is absent in the absorption spectra of the pure CuMOM (Fig. 2a) that does not bear any AgNPs and is expected to share a similar optical feature to the plain AgMOM, thus this signal at ~460 nm is likely an indication of the plasmon excitation of the AgNPs in the Ag–AgMOM.

The analysis of the decay dynamics at 460 nm for AgNPs, Ag–AgMOM (3 h) and Ag–AgMOM (28 h) (Supplementary Fig. 59b and Figure 5a-c) show strong positive signal peaking at around 460 nm was observed in the TA spectrum of pure AgNPs, which decays single exponentially (2.5 ps), possibly due to the photoinduced absorption as previously reported^43^. In contrast, the negative (bleaching) signals of Ag–AgMOM samples at 460 nm, which is related to plasmonic excitation of AgNPs therein, decay in a few ps timescales. The bleaching signal for the Ag–AgMOM (3 h) sample decays very fast (~100 fs), followed by a slow growth (~4 ps) of a positive signal that persists until 20 ps, similar to the pure AgNPs. However, the Ag–AgMOM (28 h) sample shows a slower single-exponential decay (~2 ps) of the bleach to zero (without any obvious positive signal that corresponds to the charge induced by free AgNPs). This possibly indicates a more efficient charge carrier (electron and/or hole) transfer from the AgNPs to AgMOM in the Ag–AgMOM (28 h) than in the Ag–AgMOM (3 h). This is also the underlying reason for a stronger bleaching signal at 460 nm relative to the main bleaching signal (~419 nm) in the Ag–AgMOM (28 h) sample (Supplementary Fig. 59). Besides, the transient time traces at 419 nm that corresponds to the strongest exciton bleaching feature display much faster bleaching recovery for the Ag–AgMOM (28 h) than for the Ag–AgMOM (3 h) sample (Fig. 5d). This supports the stronger exciton-plasmon coupling in the Ag–AgMOM (28 h) than in the Ag–AgMOM (3 h), leading to much faster exciton bleaching recovery in the Ag–AgMOM (28 h)^39^.

The in-situ XPS technique combined with light illumination was introduced to monitor the electron/exciton transfer in the Ag–AgMOM heterojunction. Upon 420 nm LED light irradiation, a shift of Ag^(0)^ peaks towards higher binding energies (ca. 0.3 eV) was observed in the XPS spectra of Ag 3*d*, whereas the Ag ion peaks showed little shifts, indicative of the electron density decrease of AgNPs in Ag–AgMOM (4 h) (Fig. 5e). While O 1 *s* shifted towards lower energy by −0.4 eV, suggesting an increase in the electron density (Fig. 5f), and such shift values are comparable with literature reports^44–46^. It should be noted that there were no observable shifts for C and N peaks (Supplementary Fig. 60). The previous reports have demonstrated the feasibility of the plasmonic charge transfer process from plasmonic metals to semiconductors^37–40^. The binding energy shifts in the opposite direction in the high-resolution XPS spectra provide further evidence of the efficient charge transfer across the interface in the Ag–AgMOM^44,47^.

To support our analysis, we further did light-induced in-situ XPS measurements on Ag–AgMOM (28 h), which show more substantial plasmonic effects than the Ag–AgMOM (4 h) (see the UV–Vis spectra in Fig. 2a and Supplementary Fig. 61). In this case, upon 420 nm LED light irradiation, a much more significant shift (ca. 0.9 eV) of Ag(0) peaks towards higher binding energies was observed in the XPS spectra of Ag 3*d* (Supplementary Fig. 62a). Meanwhile, the O 1*s* peak shifted towards lower energy by −0.8 eV, suggesting an increase in the electron density (Supplementary Fig. 62b). No considerable shifts were observed for C and N peaks (Supplementary Fig. 63). To further verify it, we synthesized AgNPs of a similar size (~20 nm) and surrounded by the same PVP (as the Ag–AgMOM) as a control sample. The TEM image confirmed the crystal structure and surface ligand of AgNPs (Supplementary Fig. 48). The light-induced in-situ XPS measurement of AgNPs was conducted under the same condition as the Ag–AgMOM samples (Supplementary Fig. 64). It can be seen that the C 1*s* and O 1*s* peaks (mainly from the PVP) did not show any binding energy shift. Although the Ag 3*d* peak showed a shift towards lower binding energies, it was rather small (ca. 0.2 eV). However, we can see that the peak shift direction of the Ag 3*d* peaks is opposite to that of the Ag–AgMOM under light irradiation.

DFT calculations reveal that the highest occupied valence band (HOVB) is spin-up and localized on the AgO_4_ linkers (Fig. 5g), while the lowest unoccupied conduction band (LUCB) is spin-down and distributed across the porphyrin of TCPP (Fig. 5h). The Mulliken charges of the Ag^+^ atoms in the carboxylate bridges are determined to be +1.04, thus the as-prepared Ag–AgMOM has shown apparent charge separation, beneficial to photocatalysis. Moreover, a model of a Ag cluster with three Ag atoms connected with a AgO_4_ linker in a single layer MOM, named Ag_3_-MOM, was proposed and optimized (Supplementary Fig. 65). The Mulliken charge analysis of Ag_3_-MOM shows Ag_3_ possesses an average of +0.35 charges per atom whereas the O in MOM accepts as much as −0.8 charges per atom (additional charges from Ag bridge and polarized carbonyl), manifesting a strong interaction between AgNPs and AgMOMs^48,49^. Besides, we calculated the binding energy in DFT via energy decomposition analysis (M062X/DZP) of the Ag_3_-AgMOM fragment. We found that there is substantially additional stabilization energy of –13.9 kcal/mol resulting from the association, thus supporting our experimental observation.

Based on these findings, we propose the possible SPR-enhanced photocatalytic mechanism of the Ag–AgMOM system. As shown in Fig. 5i, upon light irradiation, strong interaction between plasmonic AgNPs and excited AgMOM boost photocatalytic H_2_ evolution^50^. Although we also believe that the metallic silver is also possible to act as a co-catalyst in our system to photo-reduce H_2_O, it does not play the dominant role based on the rather low photocatalytic activities of Ag–AgMOM (1 h) and Ag–CuMOM.

## Discussion

In summary, this paper reports the synthesis of a metal-MOM hybrid with a clean and strongly interacting interface through a simple one-step approach. Such an intimate interface is believed to result from sharing the Ag atom by two components of the heterostructure (AgNP and AgMOM). Benefiting from the clean interface, the strong Raman enhancement was observed in these heterostructures and proven by SERS. Moreover, charge transfer from the plasmonic AgNPs to AgMOM is evidenced by ultrafast TA measurements, in-situ XPS and DFT calculations. Interestingly, the generally unstable Ag node-based porphyrin MOM is stabilized by AgNPs through sharing Ag atoms. Combining all the advantages of this system, i.e., the broadened light-harvesting range of plasmonic AgNPs, efficient charge/energy transfer, elevated reduction energy levels, and fast mass diffusion in thin AgMOM, the developed Ag–AgMOM yielded significantly enhanced visible-light photocatalytic H_2_ generation rate (1025 μmol h^−1^ g^−1^) and an even higher rate of 3153 μmol h^−1^ g^−1^ under the full-spectrum irradiation. The work highlights the importance of the synergistic effect of plasmonic AgNPs and metal-organic matrix in enhancing the performance of photocatalysts. We believe it will open avenues for effectively bridging metal-organic matrix and plasmonic NPs and also understanding the underlying mechanism of electron or exciton communications between components.

## Methods

### Synthesis of Ag–AgMOM

Typically, silver trifluoroacetate (5 mg), polyvinylpyrrolidone (PVP, 5 mg) and 2,4-dipyridine (1 mg) were ultrasonically dissolved in a mixture solution of 4.5 mL of DMF and 1.5 mL of ethanol in a 20 mL vial. Then TCPP (5 mg) in 1.5 mL of DMF and 0.5 mL of ethanol were added into the silver ion solution, followed by ultrasound sonication of the mixture solution for 10 min. Subsequently, the mixture was sealed and heated in an oven at 80 °C for preset time (1, 2, 3, 4, 16, 20, 28 and 36 h). The reaction solutions were then cooled down to room temperature and the products were purified 3 times by repeated centrifugation/redispersion in ethanol. Finally, the Ag–AgMOM samples were dispersed into ethanol solution and stored at 4 °C.

### Synthesis of CuMOM or ZnMOM

Copper acetate (10 mg) or Zinc acetate (10 mg), PVP (5 mg) and 2,4-dipyridine (1 mg) were ultrasonically dissolved in a mixture solution of 4.5 mL of DMF and 1.5 mL of ethanol in a 20 mL vial. Then TCPP (5 mg) in 1.5 mL of DMF and 0.5 mL of ethanol were added into the silver ion solution, followed by ultrasonication of the mixture solution for 10 min. Subsequently, the mixture was sealed and heated in an oven at 80 °C for 24 h. The reaction solutions were then purified 3 times by repeated centrifugation/redispersion in ethanol. As a result, the CuMOM samples were obtained.

### Synthesis of Ag nanocubes

Typically, silver trifluoroacetate (5 mg) and PVP (5 mg) were ultrasonically dissolved in a mixture solution of 4.5 mL of DMF and 1.5 mL of ethanol in a 20 mL vial. Then 1.5 mL of DMF and 0.5 mL of ethanol were added into the silver ion solution, followed by ultrasonication of the mixture solution for 10 min. The mixture was sealed and heated in an oven at 80 °C for 28 h. The reaction solutions were then purified 3 times by repeated centrifugation/redispersion in ethanol.

### Synthesis of AgNPs

Typically, 0.01 M of AgNO_3_ and 0.04 M of PVP were ultrasonically dissolved in a mixture solution. Then 1 mL aqueous solution of NaBH_4_ with concentration of 0.0187 g/L was added dropwise into silver ion solution under magnetic stirring. After over-night reaction at room temperature, purification was performed by 3 times repeated centrifugation/redispersion in ethanol.

### Synthesis of Ag–CuMOM

CuMOM dispersed in ethanal was mixed with AgNPs under a preset ratio and then stirred for 1 h, followed by ultrasonication of the mixture solution for 10 min, and then the reaction solutions were purified 3 times by repeated centrifugation/redispersion in water under 7800 × *g*.

### Photocatalytic performance tests

The photocatalytic hydrogen evolution activity and stability tests of samples were performed under an enclosed condition in a 500 mL Pyrex top-irradiation reactor with a quartz cover setup. Vacuum pumping was performed for 30 min before every experiment. The reaction temperature was kept at 15 °C by water circulation. In a typical process, 5 mg of the photocatalysts were dispersed in 45 mL of water (with or without 5 mL ethanol) and 2 mL triethanolamine (TEOA) was used as a hole scavenger. The solution was irradiated under a PLS-SXE300/300UV with a power of 300 W from Perfect Light Ltd with or without a 420 nm filter. The produced H_2_ was analyzed using a gas chromatograph (GC, 7890B, Agilent Technologies) equipped with a thermal conductivity detector (TCD) and a flame ionization detector (FID).

### Low-loss EELS measurements

Scanning transmission electron microscopy was conducted using a JEOL JEM F200 operated at 200 kV acceleration voltage equipped with a GATAN Continuum ER GIF for electron energy loss spectroscopy. Using a low emission current of the cold field emission gun (FEG), a narrow FWHM of 375 meV could be reached and used for low-loss spectrum imaging. Low-loss spectra were acquired with a dispersion of 15 meV, a convergence semi-angle of 12.9 mrad and a collection semi-angle of 31.2 mrad. The background tail of the zero-loss was removed by fitting a power law to the spectra. Plasmonic maps were created by choosing an energy integration window of 0.5 eV.

### Ultrafast transient absorption measurements

A custom-built TA setup from Newport, as described elsewhere^51^, was used in this work. Briefly, a multi-pass Ti:Sapphire amplifier laser system (Libra HE+, Coherent Inc.) was employed to generate ~100 fs laser pulse at 800 nm (with 1 kHz). A fraction of the 800 nm pulse was frequency-doubled to produce a 400 nm pump pulse used in the current experiment. Another fraction (~1 μJ) of the 800 nm pulse was used to generate a broad white light probe pulse by focusing on the CaF_2_ crystal. The pump pulse was allowed to pass through a 0.5 kHz optical chopper to read out the changes in probe transmission for each cycle, while a motorized delay was placed in the probe path to provide the time delay between the two pulses. The samples dispersed in ethanol were used for the experiments.

### Electrochemical and photoelectrochemical measurements

All the electrochemical and photoelectrochemical measurements were performed on a CHI 660 C electrochemical workstation (CH Instruments) in a typical three-electrode setup using a Pt as the counter electrode, Ag/AgCl as the reference electrode and fluorine-doped tin oxide (FTO) covered by samples with loading mass of 3~5 mg cm^−2^ as a working electrode. The electrolyte was 0.1 M of Na_2_SO_4_ (pH = 6.8) aqueous solution, N_2_ bubbled for ca. 0.5 h to remove O_2_ before testing. Ohmic resistance curves were scanned from 100 kHz to 10 MHz, and the scanning bias voltage was 0.2 V. For photocurrent response experiments, an AM1.5 G solar simulator (LCS-100, Newport) served as the light source, and a photochopper was used to control the light. The potential vs. NHE at different pH values was estimated using the equation, *E*_RHE_ = *E*_Ag/AgCl_ + 0.197 + 0.0592 pH.

### X-ray absorption spectroscopy (XAS)

XAS spectra at Ag K-edge (25514 eV) were collected at 20BM beamline at the Argonne National Laboratory. A Si (111) double-crystal monochromator and focused mode were used. Harmonic rejection was facilitated by a Rh-coated mirror (2 mrad) at 26 KeV. 20%Ar and 80% N_2_ were used for all ionization chambers. Details on the beamline optics and instruments can be found elsewhere^52^. The solid sample was deposited on a Kapton tape and measured in transmission mode. Several spectra were collected for each sample to ensure reproducibility and good signal-to-noise ratios. The standard Ag foil was scanned simultaneously, between the second and third ionization chambers and used for calibration.

EXAFS oscillations were extracted using Athena code and analyzed using Arthemis software^53^. The local environment of Ag atoms was determined using the phase shift and amplitude functions of Ag–O and Ag–Ag contributions from AgO and metallic Ag, respectively. The Ag foil was analyzed first and used to determine the amplitude reduction factor (*S*_0_^2^) for the samples. Due to the natural difference between the Ag foil and the samples the photoelectron energy origin correction (∆*E*_0_) determined for the foil could not be used for the samples and had to be fitted again independently. One Debye–Waller factor (σ_i_^2^) was fitted for all the contributions to reduce the number of variables. The coordination numbers (N_i_) and distances (R_i_) were adjusted freely and independently for each sample and each path. The range used to transform the EXAFS oscillations was (k^2^ χ(k)) 3–12 and the interval where the fit was performed was | χ(R)| = 1–3 Å.

### Density-functional theory (DFT) calculations

Periodic DFT was carried out using methods implemented in Amsterdam Modelling Suite 2019 BAND module. Geometry optimization was performed using PBEsol functional with D3BJ dispersion correction and employing a quasi-Newtonian approach until the changes in the energy is less than 10^−3^ Hartree and the nuclear gradient is less than 10^−3^ Hartree/Å. Bands represent the single-determinant electronic wavefunctions as a linear combination of atom-center basis sets, for which Triple Zeta plus Polarization (TVP, numerical atomic orbitals augmented by a set of Slater Type Orbitals) was used without a frozen core. Relativistic effects were accounted for by the scalar ZORA approach^54^. The convergence criterion of self-consistent field computation was set to 5.0E−08 eV. The Bernoulli zone was sampled with a symmetric K-space grid at high numerical quality. Electronic properties were computed with a range-separated exchange-correlation functional (HSE06) with the same accuracy. The Fock percentage used for HSE06 is by default 25%. Electronic properties such as band structure *E*–*K* plot were computed with a range-separated exchange-correlation functional, HSE06, with the same accuracy as geometry relaxation calculations. Electronic property calculations were done as a single-point calculation of the PBEsol optimized geometry. For Mulliken charges^49,55^, we employed HSE06 to calculate the configuration where an additional Ag_3_ cluster was placed (and structurally optimized) on top of the Ag-carboxylate linkages.

The geometry optimization, Raman spectrum, and FTIR spectra calculations of TCPP and PVP were performed using the Gaussian 16 package^56^, at PBEPBE/lanl2dz level of theory.

### Plasmonic simulation

The electromagnetic response of AgNPs has been computed using full-wave electrodynamic simulations, performed using COMSOL Multiphysics. We have simulated the response of a spherical AgNP with a diameter of 15 nm, defining its permittivity with experimental data broadened using the Drude model. The medium is set as having a constant diffractive index of *n* = 1.7, accounting for the dielectric screening of Ag-porphyrin MOM.

### Reporting summary

Further information on research design is available in the Nature Portfolio Reporting Summary linked to this article.

## Supplementary information

Supplementary Information

Reporting Summary

## Data Availability

Source data are provided with this paper.

## Source data

Source Data

## Code availability

We have used code for data analysis: Athena code (https://princetonuniversity.github.io/Athena-Cversion/), for details of the Athena code, please visit the website.
