# Supplementary material for: Silver nanoparticle enhanced metal-organic matrix with interface-engineering for efficient photocatalytic hydrogen evolution
Source: Nat Commun. 2023 Feb 1;14:541. doi: 10.1038/s41467-023-35981-8 (PMC9892045; doi:10.1038/s41467-023-35981-8)
Supplement: Supplementary file 1 — Supplementary Information [file 41467_2023_35981_MOESM1_ESM.pdf]

Supplementary Information

Silver Nanoparticle Enhanced Metal-organic Matrix with Interface-engineering for Efficient Photocatalytic Hydrogen Evolution

Yannan Liu[1,2], Cheng-Hao Liu[3], Tushar Debnath[4], Yong Wang[1], Darius Pohl[2,5], Lucas V. Besteiro[6], Debora Motta Meira[7,8], Shengyun Huang[1], Fan Yang[9], Bernd Rellinghaus[2,5], Mohamed Chaker[1], Dmytro F. Perepichka[3] & Dongling Ma[1,*]

[1]Énergie Matériaux et Télécommunications, Institut National de la Recherche Scientifque (INRS) 1650 Boul. Lionel-Boulet, Varennes, Quebec, J3X 1S2, Canada

[2] Currently at Center for Advancing Electronics Dresden (Cfaed), Technische Universität Dresden, 01062, Dresden, Germany

[3] Department of Chemistry, McGill University, 801 Sherbrooke Street West, Montreal, Quebec, H3A 0B8, Canada

[4] Chair for Photonics and Optoelectronics Nano-Institute Munich Department of Physics, Ludwig-Maximilians-University, Königinstr. 10, 80539, München, Germany

[5] Dresden Center for Nanoanalysis (DCN), 01062, Dresden, Germany

[6] CINBIO, Universidade de Vigo, 36310 Vigo, Spain

[7] CLS@APS sector 20, Advanced Photon Source, Argonne National Laboratory, 60439, Lemont, IL, USA.

[8] Canadian Light Source Inc., Saskatoon, SK, S7N 2V3, Canada.

[9] Department of Materials Science and Engineering, Stanford University, Stanford, CA, 94305, USA

*Correspondence and requests for materials should be addressed to D.M. (e-mail: ma@emt.inrs.ca).

Table of Contents

1. Supplementary Materials.

2. Instrumentation and Methods.

3. Results and Discussion.

4. Supplementary References.

1. Supplementary Materials.

N,N-Dimethylformamide (DMF), ethanol (≥99.0%), polyvinylpyrrolidone (PVP), silver trifluoroacetate, acetonitrile, trifluoroacetic acid (TFA), 4,4′−Dipyridyl and triethanolamine (≥99.0%) were obtained from Sigma Company and used without further purification. Tetrakis (4-carboxyphenyl) porphyrin (TCPP) was purchased from TCI America. All of the water used in this work was ultrapure water.

2. Instrumentation and Methods.

Transmission Electron Microscopy (TEM)

TEM and high-resolution TEM (HR-TEM) were carried out in a JEOL JEM-2100F at a voltage of 200 kV. The TEM samples were prepared by dropping sample solutions onto lacey carbon-coated copper or nickel grids. Energy-dispersive X-ray spectrometry (EDS) analysis and selected area electron diffraction (SAED) were also conducted in the TEM system to probe the distribution of chemical elements or crystal diffraction in Ag-AgMOM.

Atomic Force Microscopy (AFM)

The topography images and the corresponding height measurements of Ag-AgMOM samples were obtained on MultiMode 8 AFM (Bruker) in a tapping mode. Ag-AgMOM samples were placed on the Highest Grade V1 AFM Mica Discs (12 mm, Ted Pella, Inc.). The flatness of the mica discs is smaller than 1 nm, and the hardness is 80-105 shore test.

Ultraviolet-visible (UV-Vis) Absorption Spectroscopy

The UV-Vis absorption spectra of solution samples were taken on a Cary 5000 UV-Vis spectrophotometer (Varian) in the range from 300 to 700 nm at 25 ºC. A 1 cm quartz cuvette was used for the measurements.

Diffuse Reflectance Spectra (DRS)

DRS measurements of powder samples were performed in a UV-Visible-PIR spectrometer (Perkin Elmer, Lambda 750) with an integrating sphere. The bandgap (Eg) of Ag-AgMOM and TCPP samples was determined from DRS and the Tauc plots were derived from these measurements.

Raman Spectra

The Raman spectra of TCPP, PVP and Ag-AgMOM samples were acquired by a Raman microscope (Renishaw, inVia Reflex), including an optical microscope (x5, x20 and x50) coupled to a Raman spectrometer. The excitation laser wavelength was 532 nm. The optical microscope is used to enlarge and identify the sample areas, and the Raman spectrometer measures the vibrations under excitation.

Fourier-transform Infrared Spectroscopy (FTIR)

FTIR spectra of samples were recorded on a Thermo Scientific spectrometer to identify the structure of samples from 400 to 4000 cm^{-1}.

Neutron Activation Analysis (NAA)

NAA was carried out using a SLOWPOKE nuclear reactor to detect the concentration of Ag element in Ag-AgMOM samples.

Inductively Coupled Plasma Optical Emission Spectroscopy (ICP-OES)

ICP-OES measurements were conducted on an Agilent Technologies, 5100 spectrometer for the determination of Ag mass percentage in Ag-AgMOM and Ag-CuMOM samples. The method consists of ionizing the sample by injecting an argon plasma (temperature ca. 6000K). The standard Ag plasma solution (Specpure®) was purchased from Alfa Aesar. The Ag-AgMOM solution was prepared by acidic digestion of Ag-AgMOM in 5% of HNO_3 water solution. A clear aqueous solution containing the dissolved sample was obtained at the end. The solution was then injected into the plasma in the form of a fine aerosol, generated by a pneumatic device (nebulizer).

X-ray Photoelectron Spectroscopy (XPS)

XPS measurements of samples were performed on a VG Escalab 220i-XL equipped with an Al-KαX-ray source. The obtained spectra were calibrated using the C 1s peak at the position of 284.6 eV. Operando XPS was conducted by combining it with a 420 nm light-emitting diode (LED, 20W) during measurements.

Powder X-ray Diffraction (PXRD)

PXRD (PANalytical X'Pert MRD) equipped with a Cu Kα radiation source (λ = 0.15406 nm) was used to explore the crystal structures of samples. PXRD analysis was conducted using the Reflex module in Materials Studio 2019. Two-dimensional PXRD simulation was conducted using numerical integration of real space of a finite system reported by literature.[1] All 3D simulated PXRD patterns are generated with the default setting (i.e., no broadenings/shifts, standard Cu source) in Materials Studio.

Brunauer-Emmett-Teller (BET) measurements

Nitrogen adsorption-desorption isotherm measurements were conducted on Micromeritics Instrument Corporation TriStar II 3020 3.02 TriStar II 3020 from 0 to 1 atm at 77 K. The Brunauer-Emmett-Teller (BET) method was utilized to calculate the specific surface areas. And

non-local density functional theory (NLDFT) model was used to determine the pore sizes and volumes. The pore size distribution of simulated AgMOM was performed using software of PoreBlazer (v4.0).

3. Results and Discussion.

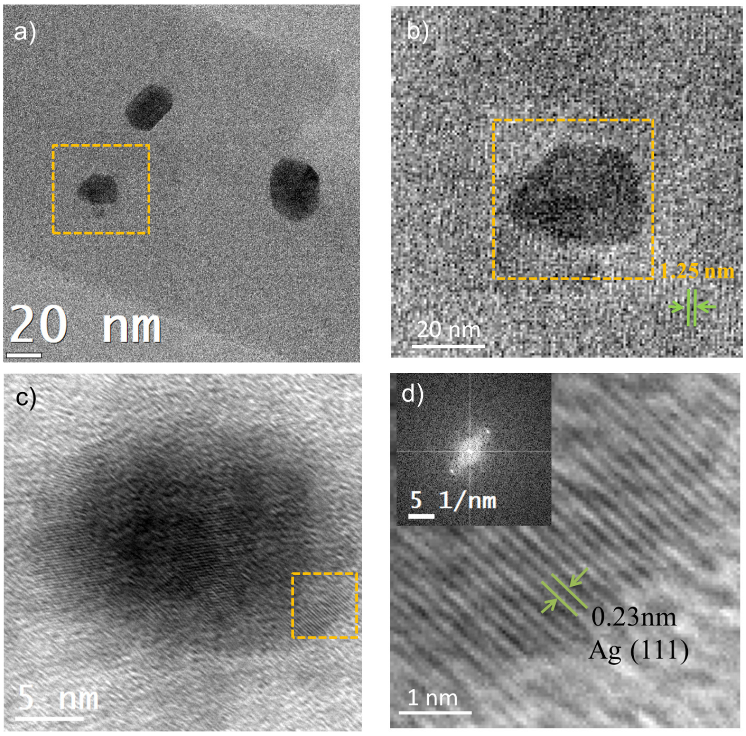

Supplementary Fig. 1 (a,b) Enlarged TEM images of Ag-AgMOM; the lattice of AgMOM can be observed. (c,d) HR-TEM images and crystal lattice of a single AgNP on MOM. Inset in (d) is the fast Fourier transform image of the AgNP.

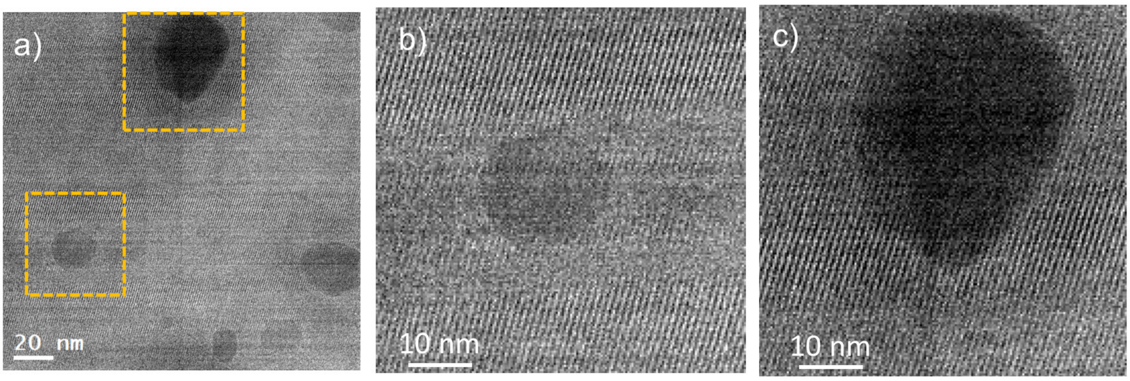

Supplementary Fig. 2 (a) TEM image of Ag-AgMOM and (b,c) HR-TEM images of Ag-AgMOM highlighted in a). The clear lattice of the AgMOM can be observed around Ag NPs, suggesting an ordered structure of the AgMOM.

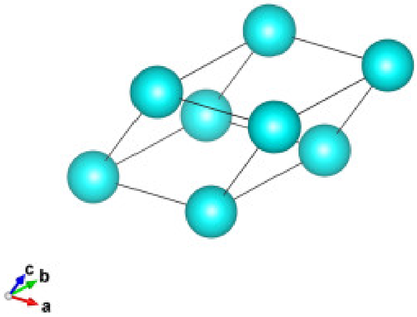

Supplementary Fig. 3 The primitive cubic crystal structure model of AgNPs in Ag-porphyrin MOM with the space group of $Fm\bar{3}m$.

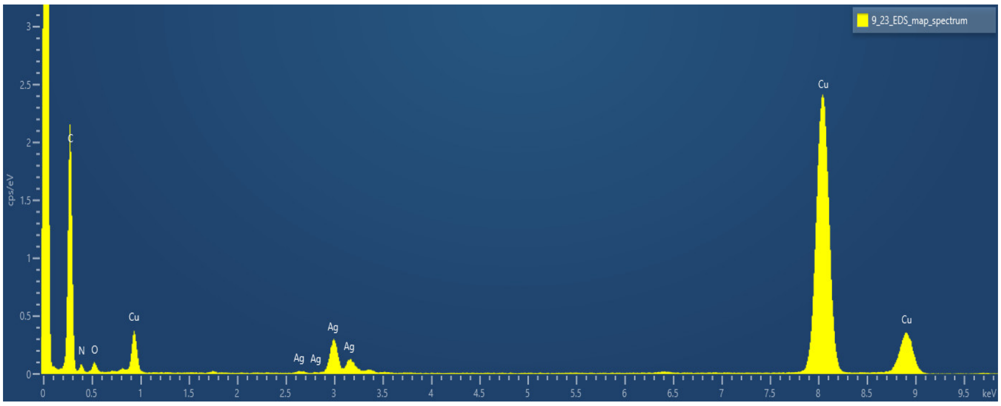

Supplementary Fig. 4 Energy-dispersive X-ray spectroscopy **(**EDS) spectrum of Ag-AgMOM (Cu signal comes from the Cu grid).

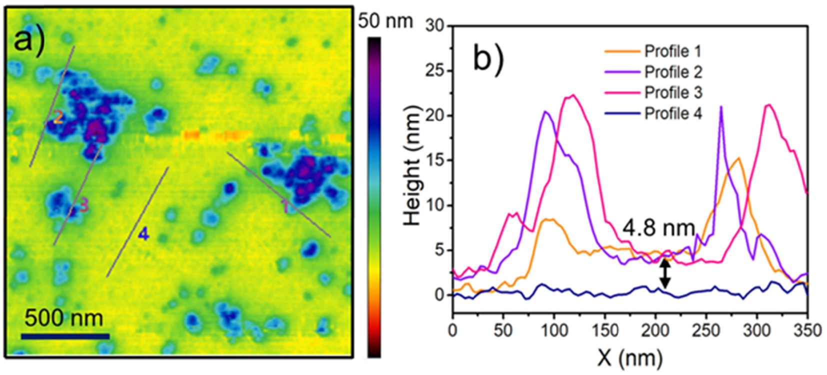

Supplementary Fig. 5 (a) AFM image of Ag-AgMOM on the highest grade AFM mica disc. (b) Height profiles of the AgMOM in Ag-AgMOM.

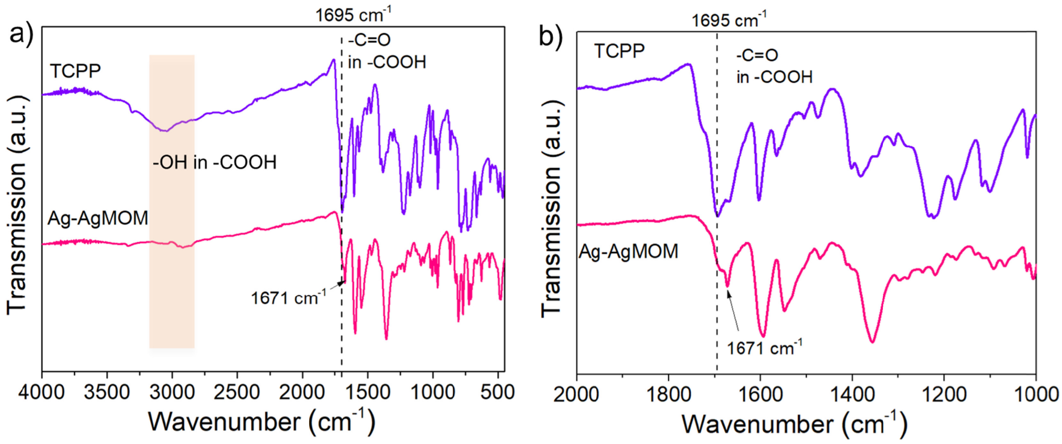

Supplementary Fig. 6 (a) Experimental FTIR spectra of Ag-AgMOM and TCPP. (b) is the enlarged spectra range from 1000 ~ 2000 cm^{-1}.

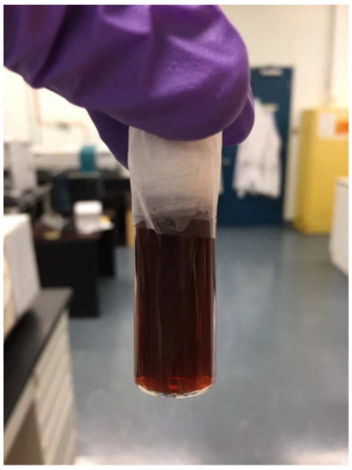

Supplementary Fig. 7 Photograph of dispersion of Ag-AgMOM in water after 1 month storage at 4 °C, confirming its long term colloidal stability.

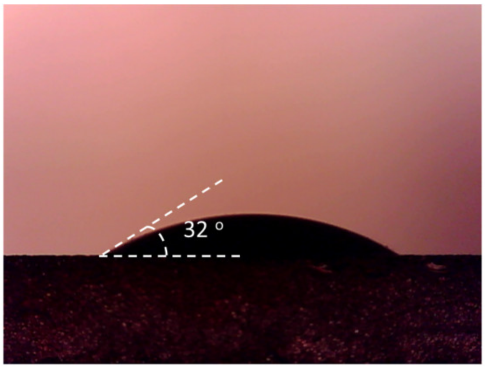

Supplementary Fig. 8 Water contact angle of the Ag-AgMOM film.

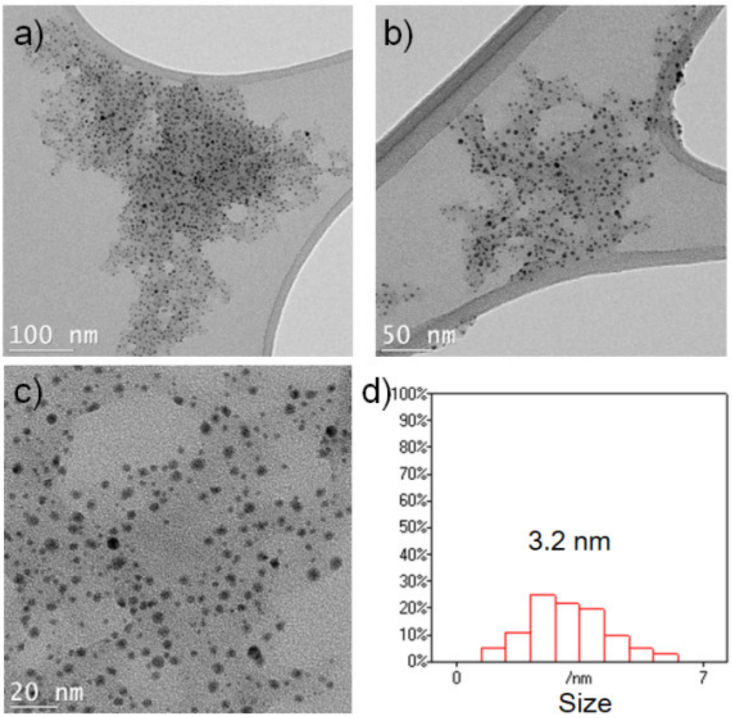

Supplementary Fig. 9 (a-c) Typical TEM images of Ag-AgMOM and (d) the size distribution histogram of AgNPs synthesized under the conditions of m_{Ag+}/m_{TCPP}=1:1 and reaction time of 1 h.

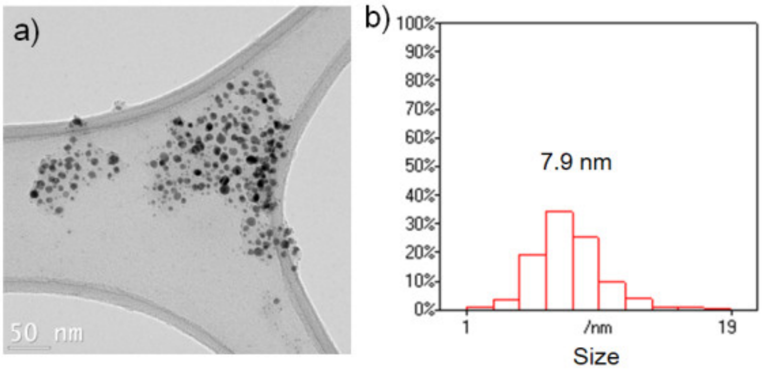

Supplementary Fig. 10 (a) Typical TEM image of Ag-AgMOM and (b) the size distribution histogram of AgNPs synthesized under the conditions of m_{Ag+}/m_{TCPP}=1:1 and reaction time of 4 h.

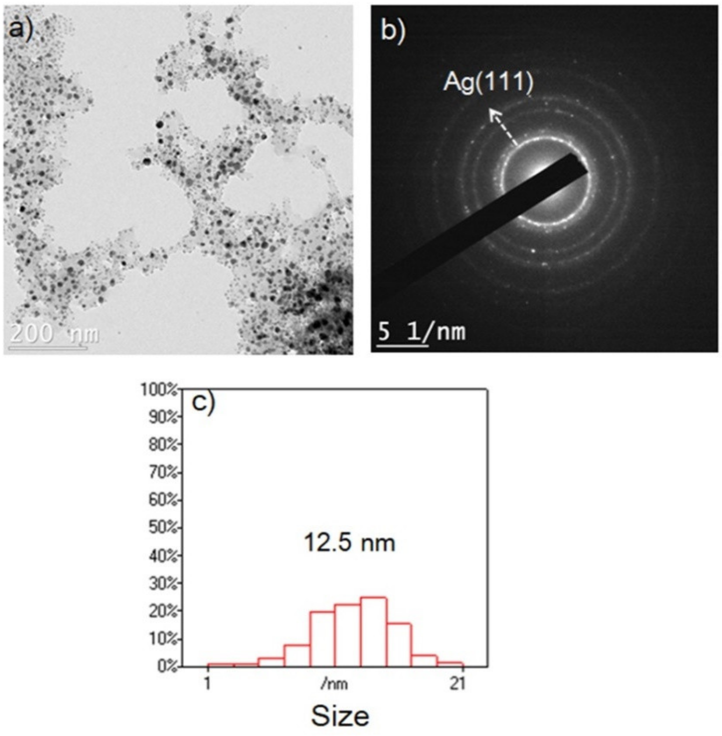

Supplementary Fig. 11 (a) Typical TEM images, (b) SAED of Ag-AgMOM, and (c) the size distribution histogram of AgNPs synthesized under the conditions of m_{Ag+}/m_{TCPP}=1:1 and reaction time of 20 h.

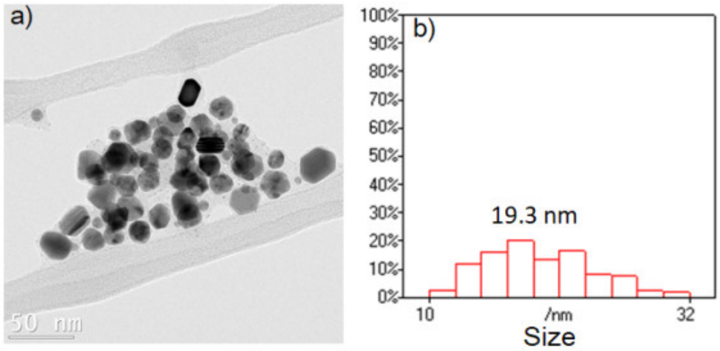

Supplementary Fig. 12 (a) Typical TEM image of Ag-AgMOM and (b) the size distribution histogram of AgNPs synthesized under the conditions of m_{Ag+}/m_{TCPP}=1:1 and reaction time of 36 h.

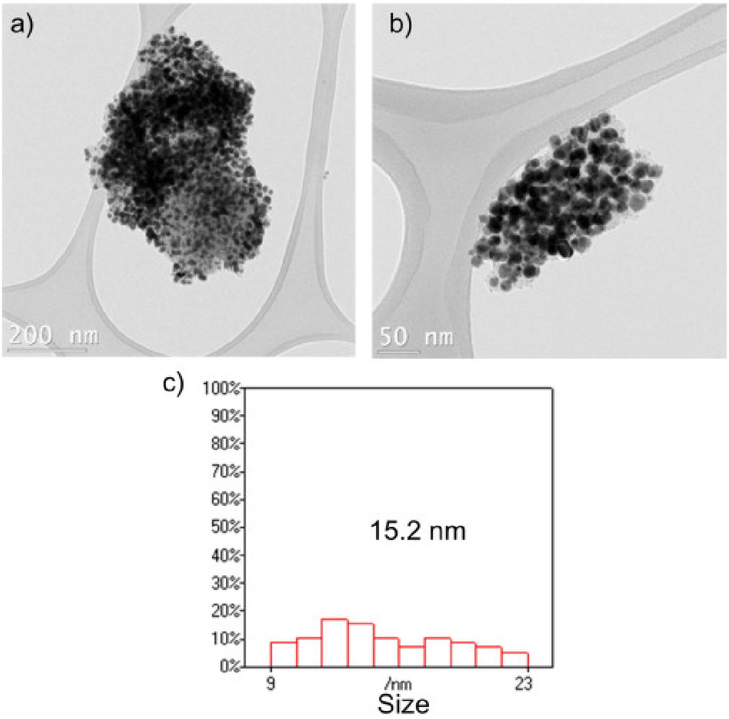

Supplementary Fig. 13 (a,b) Typical TEM images of Ag-AgMOM and (c) the size distribution histogram of AgNPs synthesized under the conditions of m_{Ag+}/m_{TCPP}=2:1 and reaction time of 28 h.

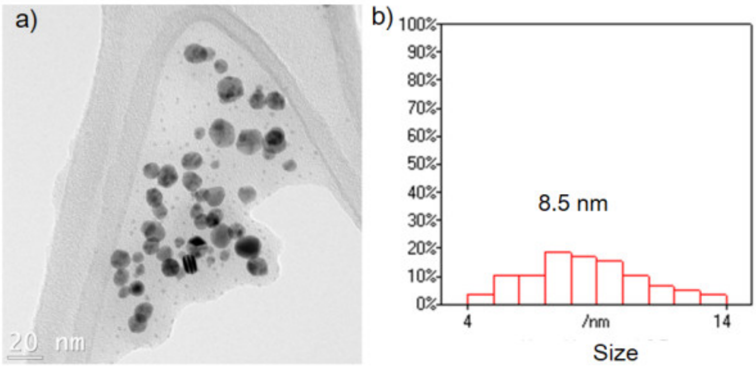

Supplementary Fig. 14 (a) Typical TEM image of Ag-AgMOM and (b) the size distribution histogram of AgNPs synthesized under the conditions of m_{Ag+}/m_{TCPP}=1:4 and reaction time of 28 h.

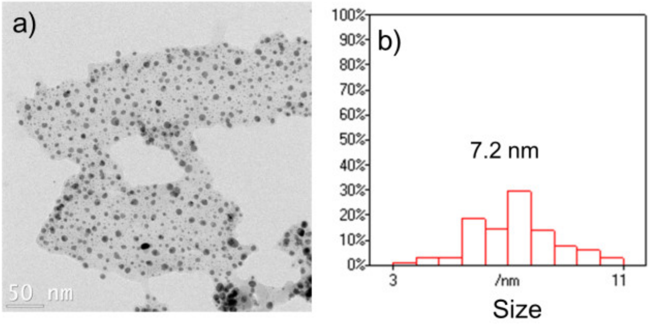

Supplementary Fig. 15 (a) Typical TEM image of Ag-AgMOM and (b) the size distribution histogram of AgNPs synthesized under the conditions of m_{Ag+}/m_{TCPP}=1:2 and reaction time of 4 h.

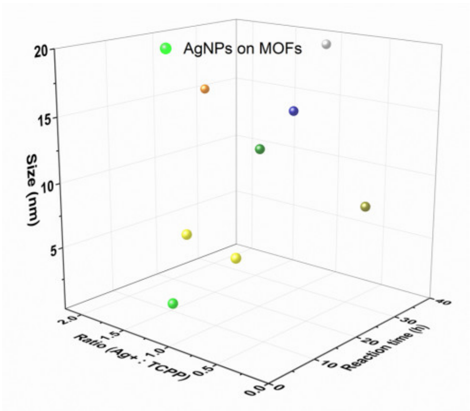

Supplementary Fig. 16 The statistically measured average size of AgNPs on Ag-porphyrin MOM synthesized under different reaction time and feeding ratios, without using any regulating agent.

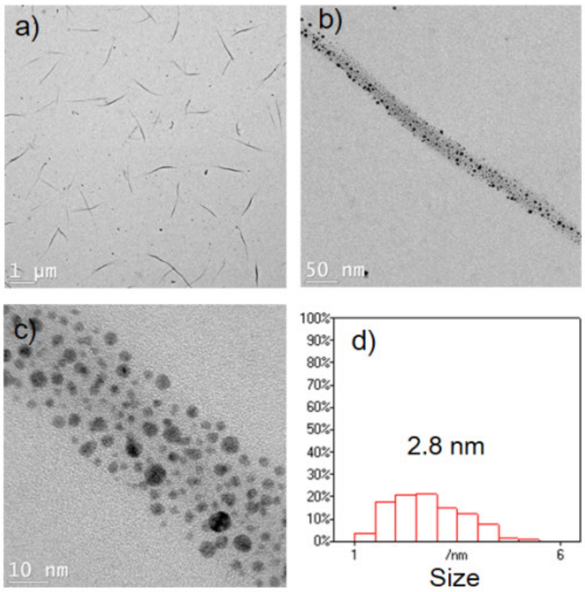

Supplementary Fig. 17 (a-c) Typical TEM images of Ag-AgMOM and (d) the size distribution of AgNPs under m_{Ag+}/m_{TCPP}=1:1, reaction time of 28 h with 100 μL TFA as a regulating agent.

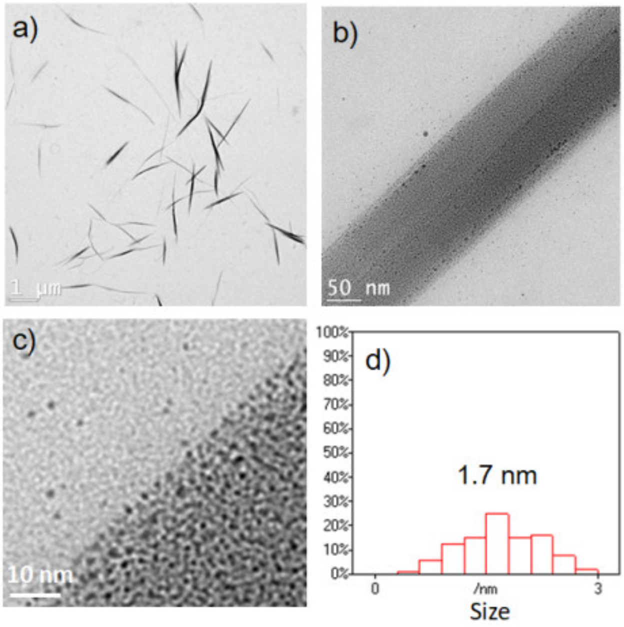

Supplementary Fig. 18 (a-c) Typical TEM images of Ag-AgMOM and (d) the size distribution histogram of AgNPs synthesized under the conditions of m_{Ag+}/m_{TCPP}=1:1, reaction time of 28 h and with the introduction of 200 μL TFA.

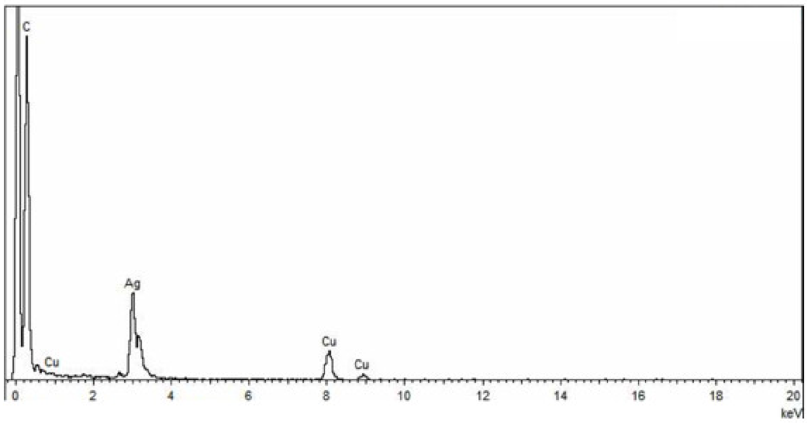

Supplementary Fig. 19 EDS spectrum of Ag-AgMOM synthesized under the conditions of m_{Ag+}/m_{TCPP}=1:1, reaction time of 28 h and with the addition of 200 μL TFA (Cu comes from the TEM grid).

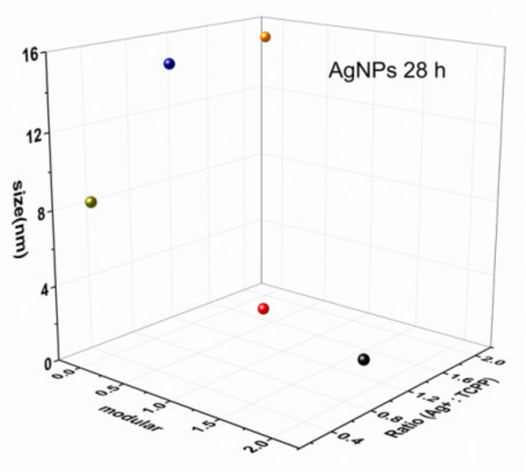

Supplementary Fig. 20 The statistically average size of AgNPs on MOM synthesized under different feeding ratios of m_{Ag+} to m_{TCPP} and amounts of TFA at the same reaction time of 28 h.

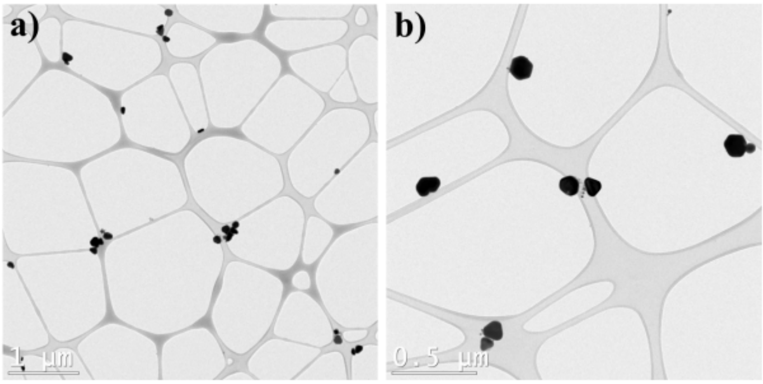

Supplementary Fig. 21 (a,b) Typical TEM images of Ag nanocubes, which was synthesized under the same condition as that of Ag-AgMOM (28 h), except for the absence of TCPP ligands.

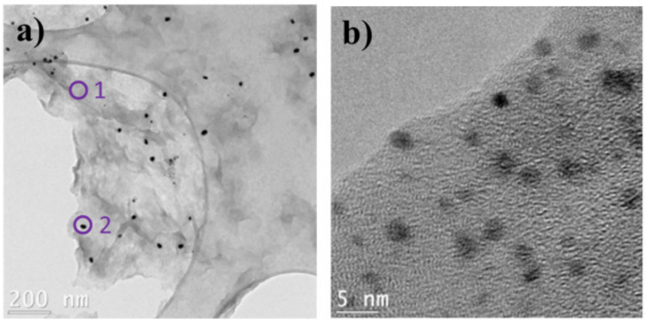

Supplementary Fig. 22 (a,b) Typical TEM images of Ag-CuMOM synthesized under the conditions of $m_{Cu2+}/m_{Ag+}/m_{TCPP}$=1:1:1 and reaction time of 28 h. Circles 1 and 2 in (a) denote the areas from which EDS was taken, as shown below in Supplementary Fig.23.

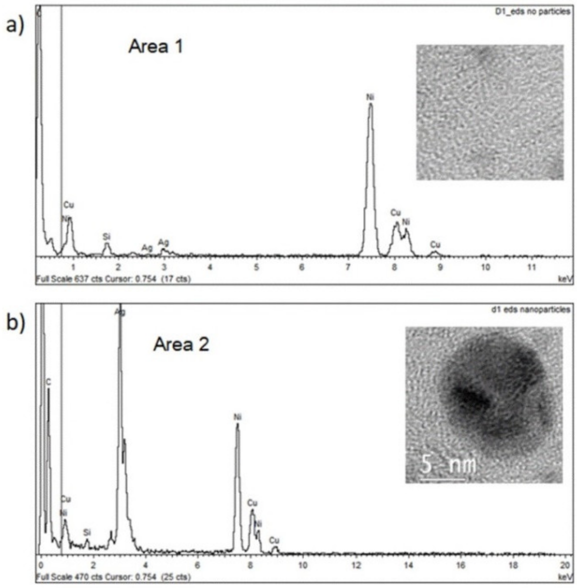

Supplementary Fig. 23 EDS spectra of Ag-CuMOM taken from (a) the area 1 and (b) the area 2 in Supplementary Fig. 22 (a).

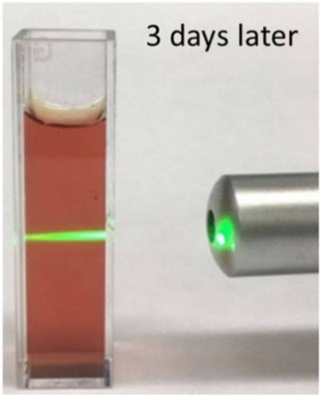

Supplementary Fig. 24 Photograph of Ag-CuMOM in ethanol under green laser illumination.

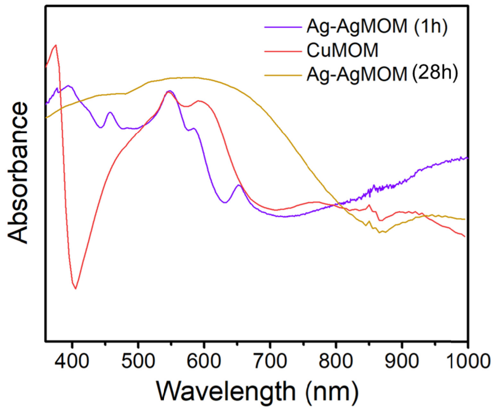

Supplementary Fig. 25 UV-Vis spectra of solid CuMOM, Ag-AgMOM (1h) and Ag-AgMOM (28h).

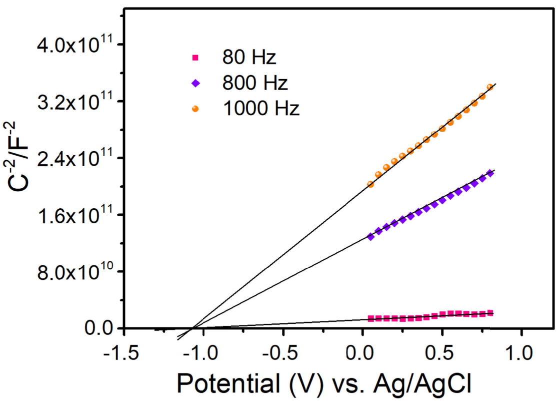

Supplementary Fig. 26 Mott-Schottky plots of CuMOM.

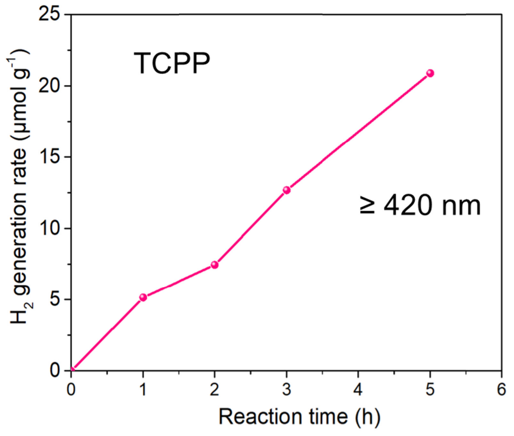

Supplementary Fig. 27 Photocatalytic performance of TCPP under 300 W irradiation with a 420 nm long-pass filter (H_2 generation rate: ~ 4 μmol h^{-1} g^{-1}).

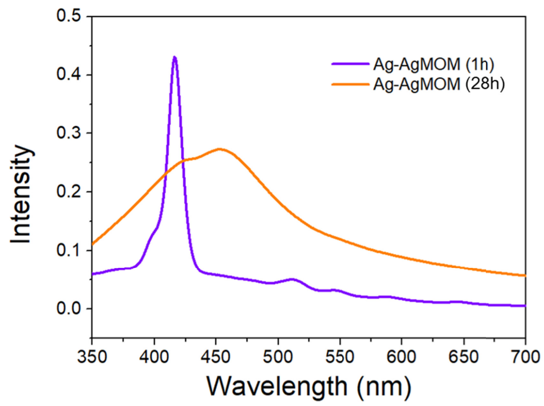

Supplementary Fig. 28 UV-Vis absorption spectra of Ag-AgMOM (28h) and Ag-AgMOM (1h) in ethanol solution.

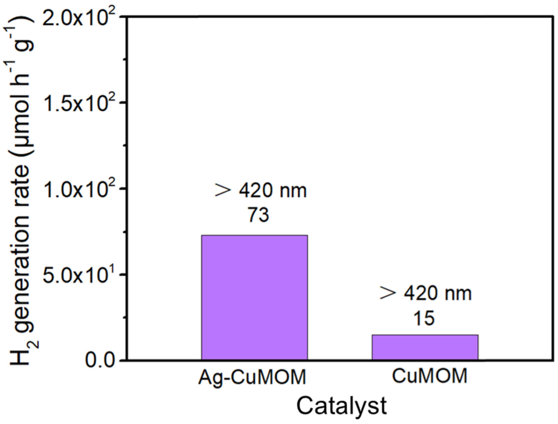

Supplementary Fig. 29 Hydrogen generation rate under 300 W irradiation with a 420 nm long-pass filter and in the presence of Ag-CuMOM and CuMOM, respectively.

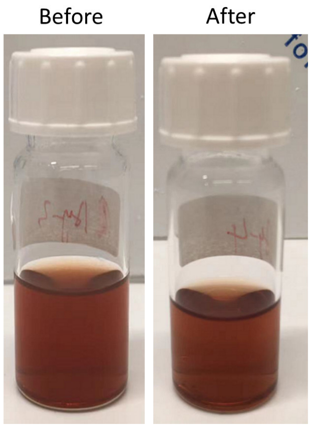

Supplementary Fig. 30 Photographs of Ag-AgMOM in solution before and after photocatalytic reaction.

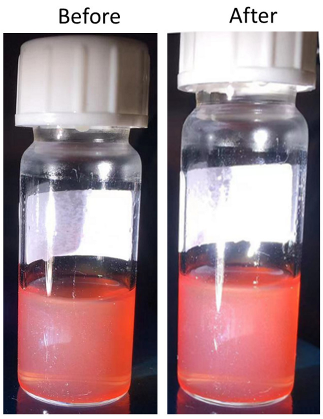

Supplementary Fig. 31 Photographs of Ag-AgMOM in solution under Xe lamp light irradiation before and after photocatalytic reaction.

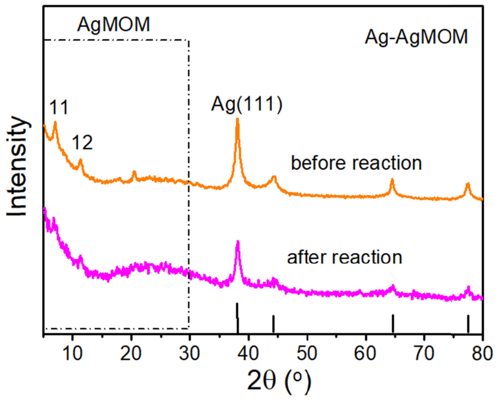

Supplementary Fig. 32 PXRD patterns of the Ag-AgMOM before and after photocatalytic reaction.

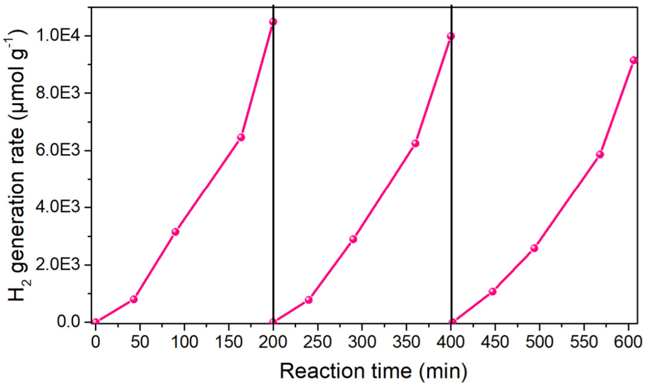

Supplementary Fig. 33 Three cycles of photocatalytic performance of Ag-AgMOM.

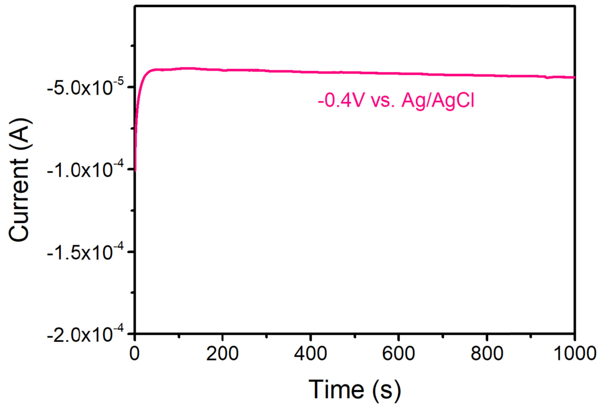

Supplementary Fig. 34 Photocurrent stability of Ag-AgMOM at a bias of -0.4 V vs. Ag/AgCl.

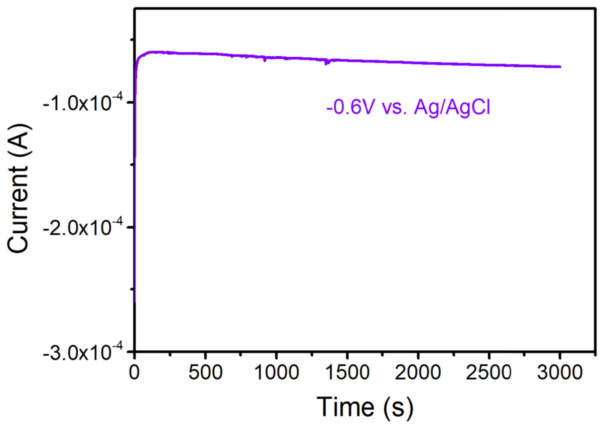

Supplementary Fig. 35 Photocurrent stability of Ag-AgMOM at a bias of -0.6 vs. Ag/AgCl.

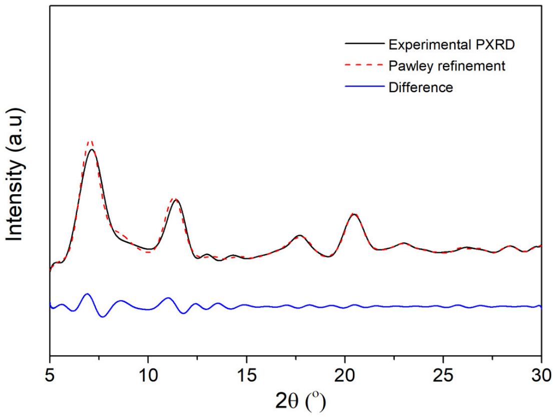

Supplementary Fig. 36 Pawley refinement of experimental PXRD of Ag-AgMOM comparing with the simulated model in the range from 2-30° (Rwp =5.20 % and Rp = 3.84%).

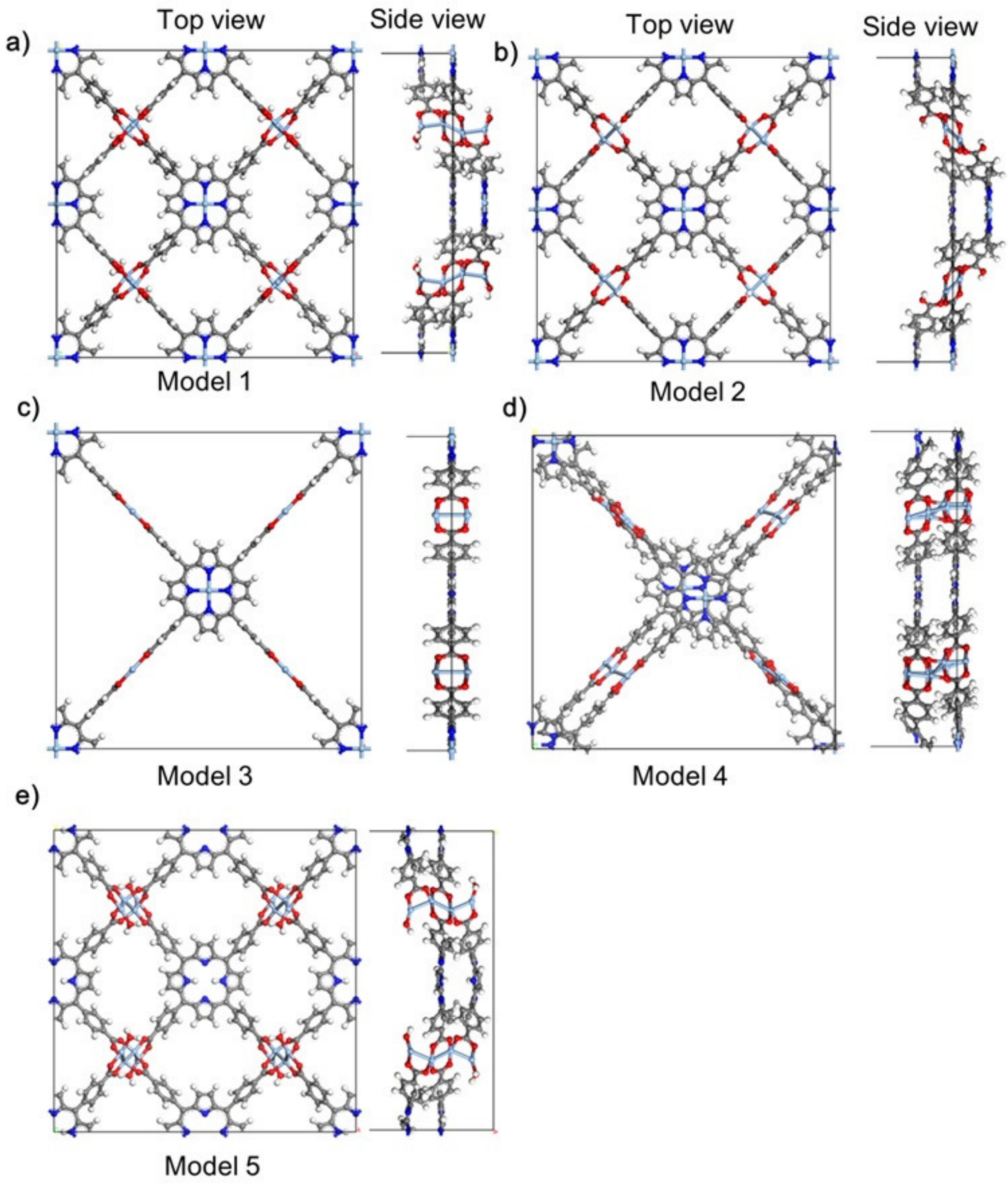

Supplementary Fig. 37 (a-e) More models optimized and considered for AgMOM. None of them have simulated PXRD patterns matching with the experimental PXRD pattern. While the simulation results of our model may also present a little mismatch with experimental data, same as most simulated PXRD[2,3], it is self-consistent and provides an overarching explanation to the observed phenomena.

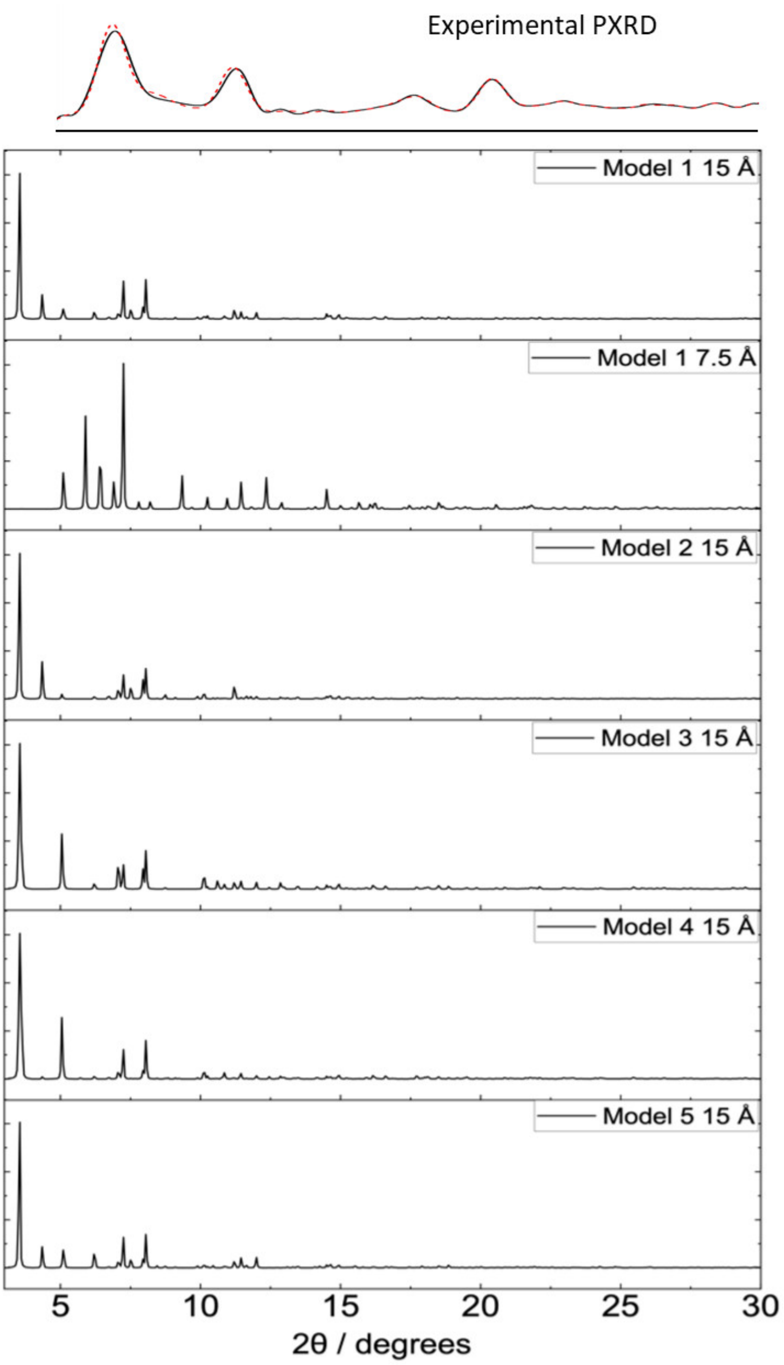

Supplementary Fig. 38 Experimental PXRD (the top one) and simulated PXRD patterns of the other models shown in Supplementary Fig. 37, under the assumption of AA-stacking with c-distance indicated in the legend.

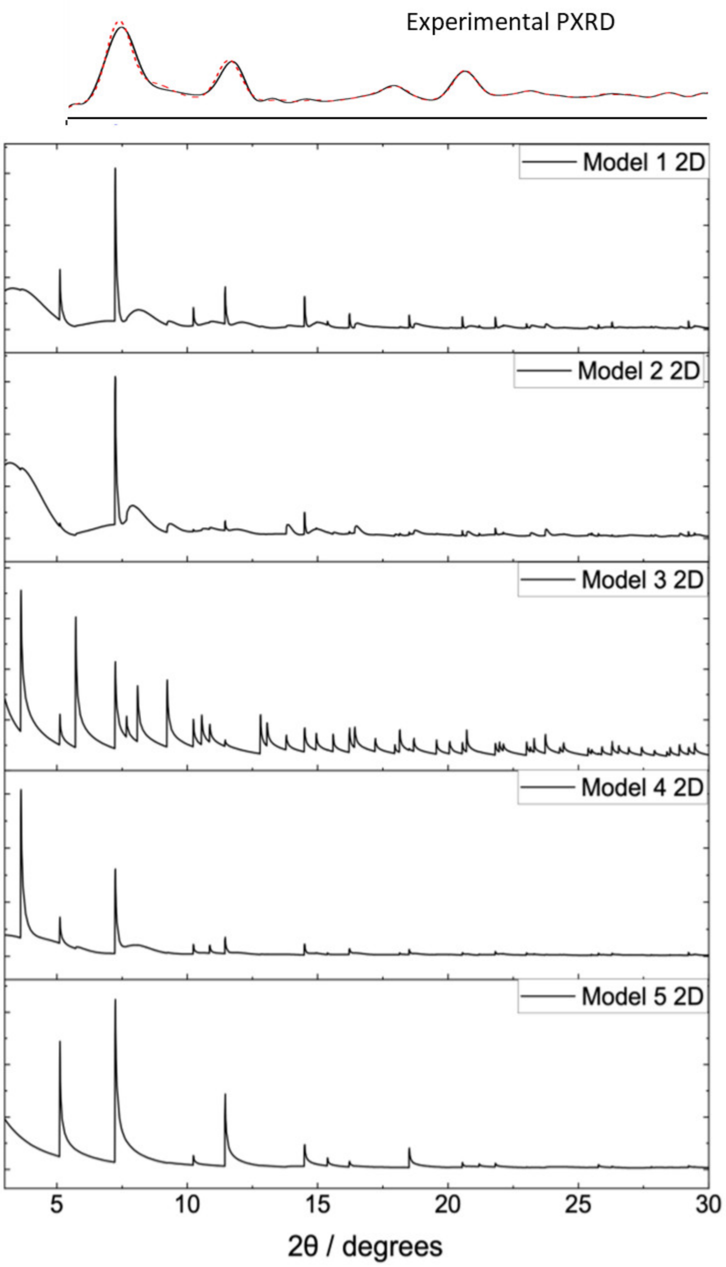

Supplementary Fig. 39 Experimental PXRD (the top one) and simulated 2D PXRD patterns of the other models shown in Supplementary Fig. 37.

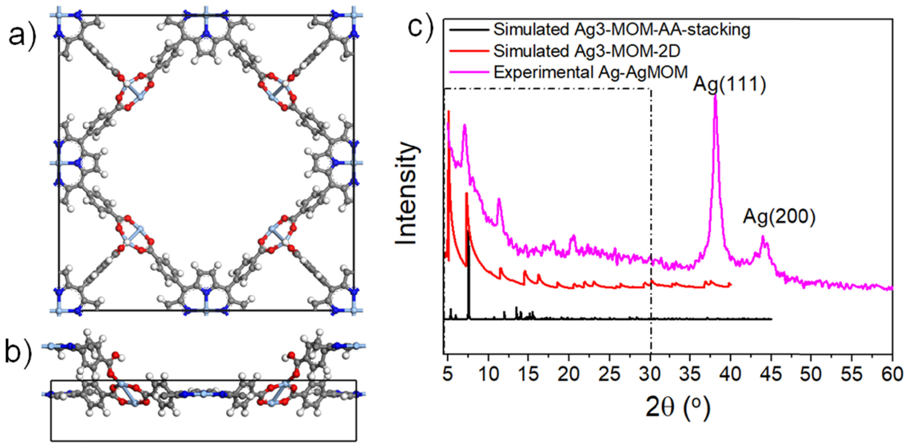

Supplementary Fig. 40 (a) Top view and (b) side view of a model of two silver atoms bonded per three carboxylates considered for AgMOM. (c) The simulated PXRD patterns in AA-stacked and in 2D comparing with the experimental PXRD pattern of Ag-AgMOM.

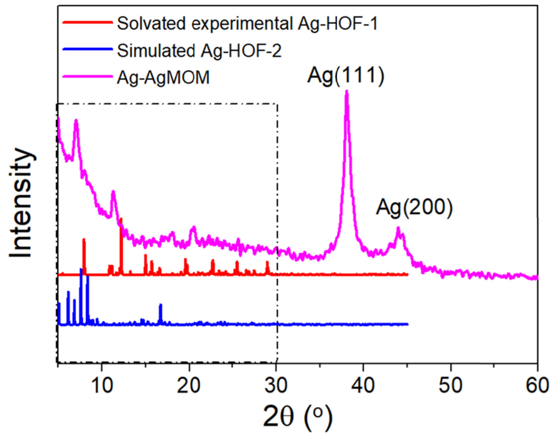

Supplementary Fig. 41 The simulated PXRD patterns of solvated experimental Ag-TCPP hydrogen-bonded structure (Ag-HOF-1 reported in *J. Porphyrins Phthalocyanines* **14**, 804 (2010).) and simulated Ag-HOF-2 comparing with the experimental PXRD pattern of Ag-AgMOM.

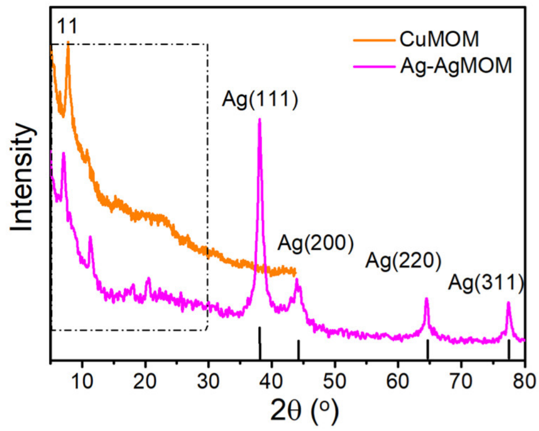

Supplementary Fig. 42 PXRD pattern comparison between Ag-AgMOM and CuMOM.

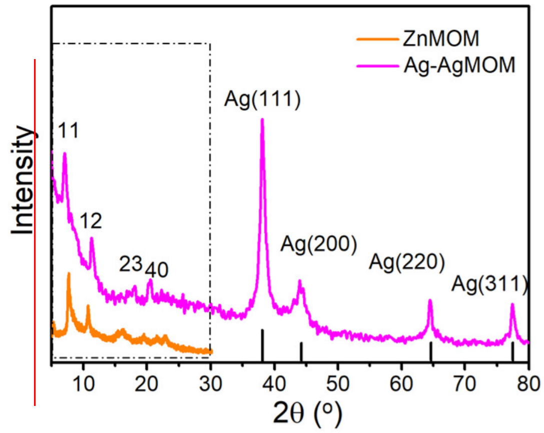

Supplementary Fig. 43 PXRD pattern comparison between Ag-AgMOM and ZnMOM.

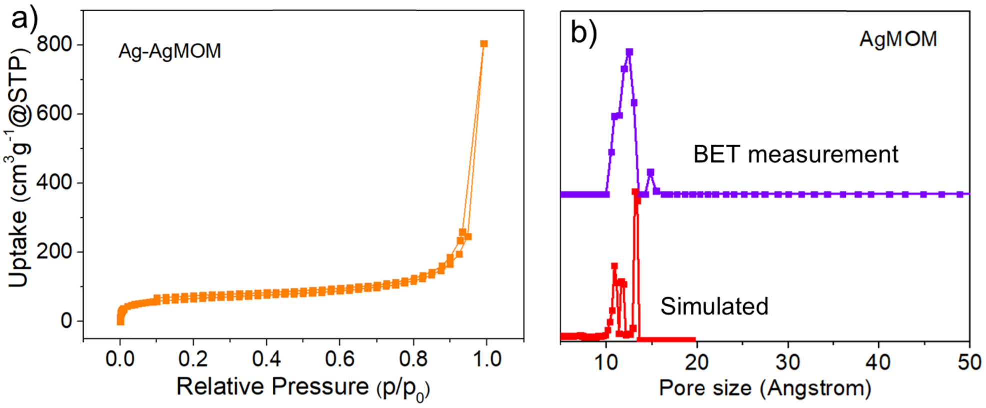

Supplementary Fig. 44 (a) N_2 adsorption and desorption isotherm curves of Ag-AgMOM. (b) The simulated pore size distribution curve of AgMOM and pore size distribution profile from BET experiment of Ag-AgMOM.

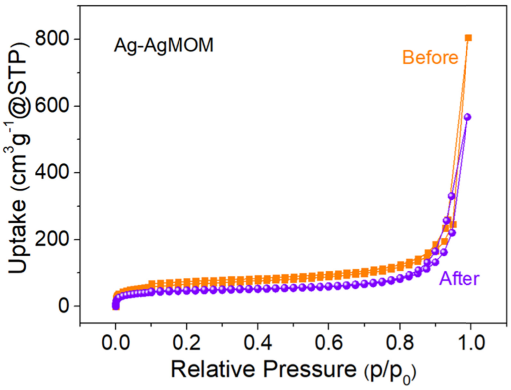

Supplementary Fig. 45 N_2 adsorption and desorption isotherm curves before and after photocatalysis under full spectrum light irradiations.

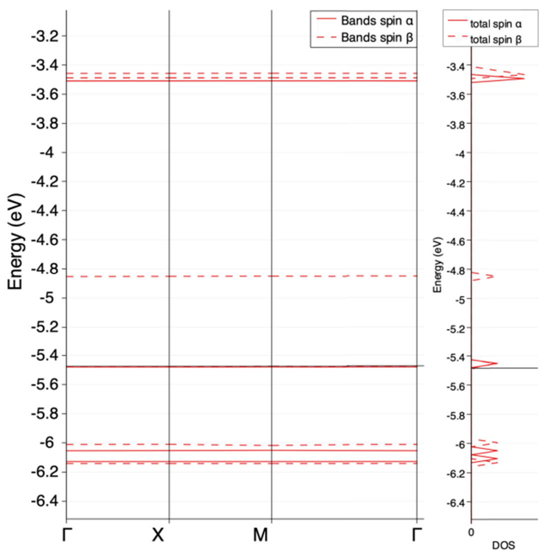

Supplementary Fig. 46 Calculated band structure and total density of states (TDOS) of AgMOM.

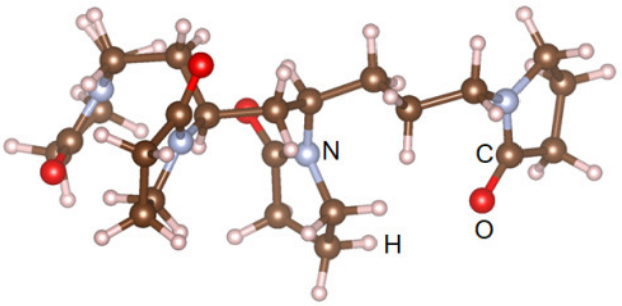

Supplementary Fig. 47 Molecular model of simplified PVP (n=4).

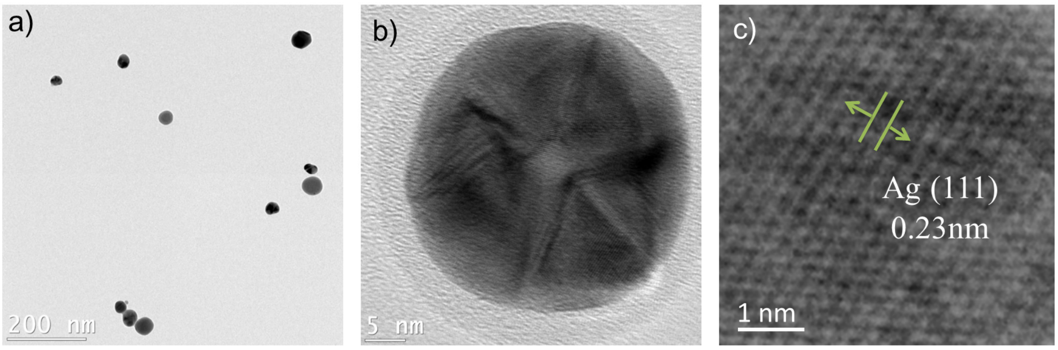

Supplementary Fig. 48 (a) TEM image of AgNPs. (b) TEM image of a single AgNP, where the surfactant layer on the surface can be observed. (c) HR-TEM image of a AgNP clearly showing lattice fringes with the spacing of 0.23 nm, in line with the Ag (111) crystal planes.

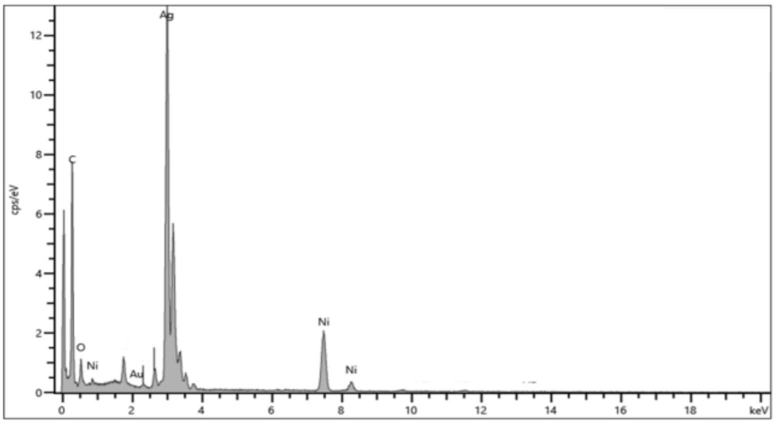

Supplementary Fig. 49 EDS spectrum of AgNPs (Ni comes from the TEM grid).

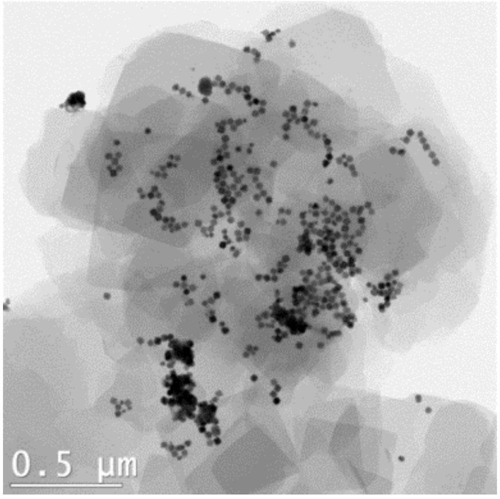

Supplementary Fig. 50 TEM image of Ag-CuMOM.

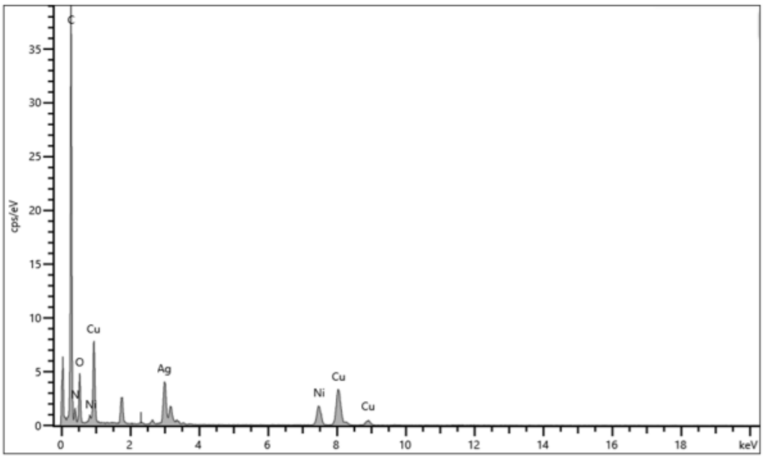

Supplementary Fig. 51 EDS spectrum of Ag-CuMOM**.**

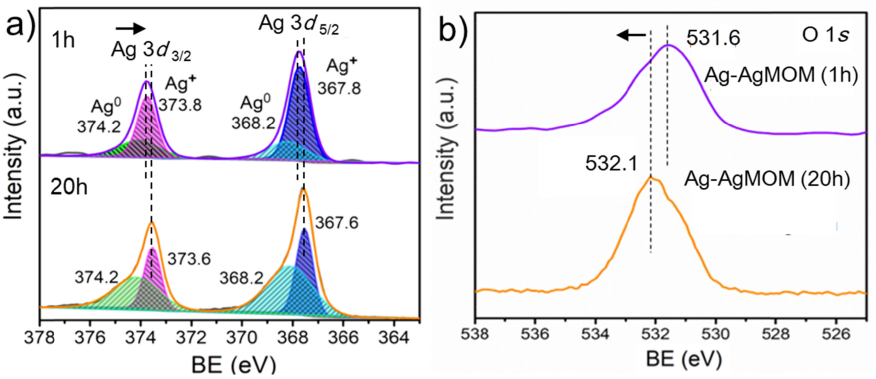

Supplementary Fig. 52 XPS peaks of (a) Ag 3$d_{3/2}$, Ag 3$d_{5/2}$ and (b) O 1s in Ag-AgMOM synthesized at the reaction time of 1h and 20h (BE: binding energy).

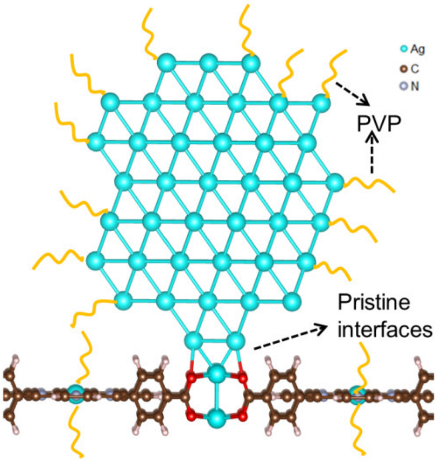

Supplementary Fig. 53 Side view of the model of one Ag NP linked with porphyrin AgMOM.

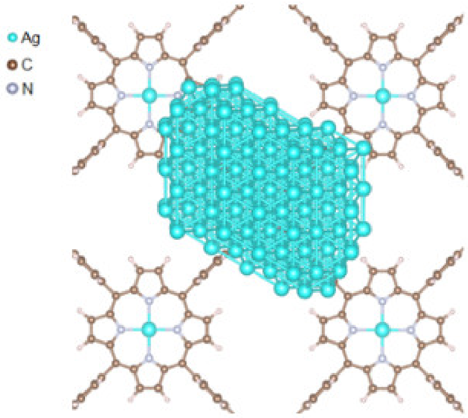

Supplementary Fig. 54 Top view of the model of one Ag NP linked with porphyrin AgMOM.

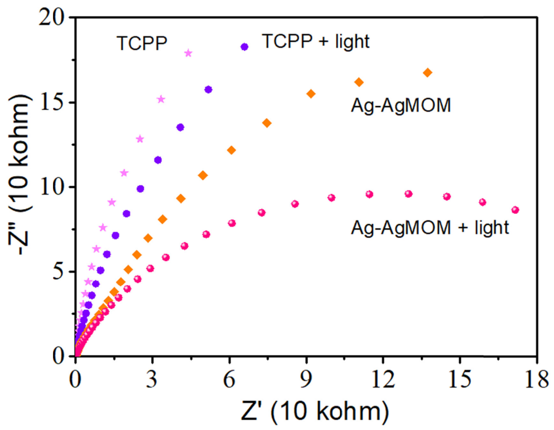

Supplementary Fig. 55 EIS spectra of TCPP and Ag-AgMOM under light or dark conditions.

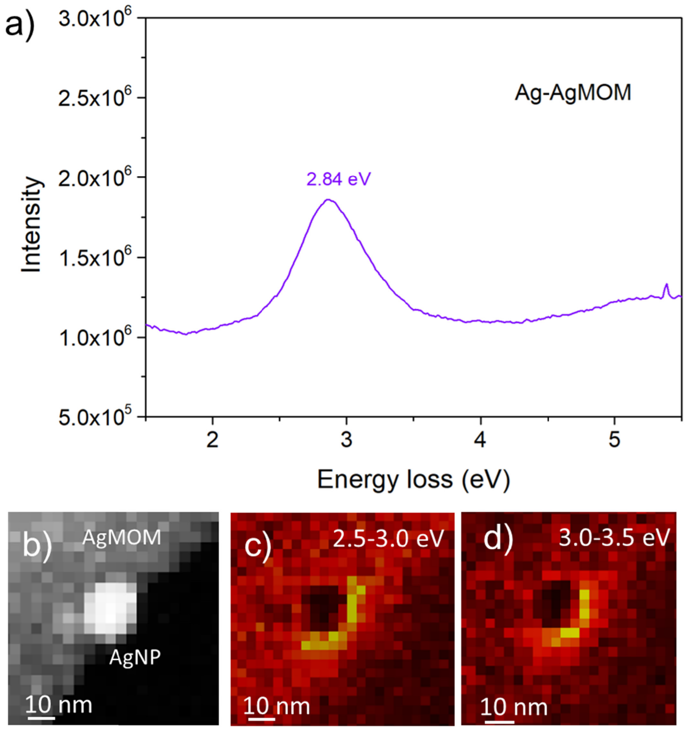

Supplementary Fig. 56 (a) EELS spectrum of Ag-AgMOM after background subtraction (zero-loss tail was removed). (b) HAADF-STEM image of Ag-AgMOM. (c,d) Plasmon mapping of Ag-AgMOM in two different energy ranges.

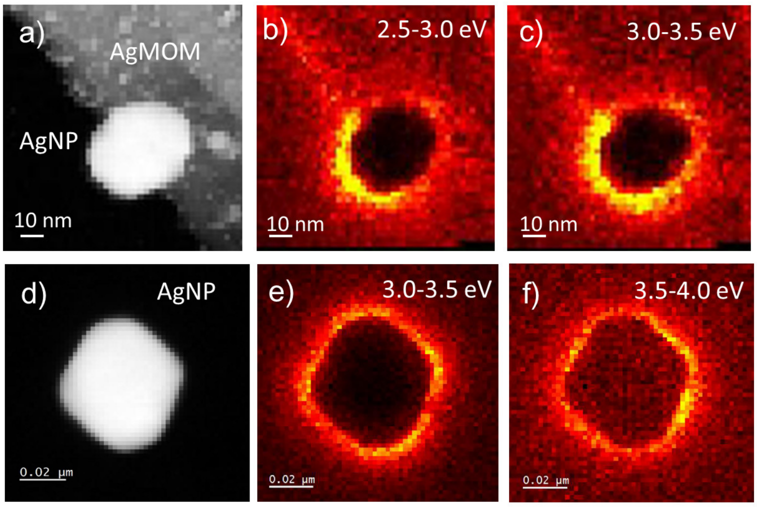

Supplementary Fig. 57 (a) HAADF-STEM image of Ag-AgMOM. (b,c) Plasmon mapping of Ag-AgMOM in two different energy ranges. (d) HAADF-STEM image of AgNP. (e,f) Plasmon mapping of AgNP in two different energy ranges. Here, pure AgNP and AgNP on AgMOM have a similar size of ca. 40 nm, larger than that shown in Supplementary Fig. 51.

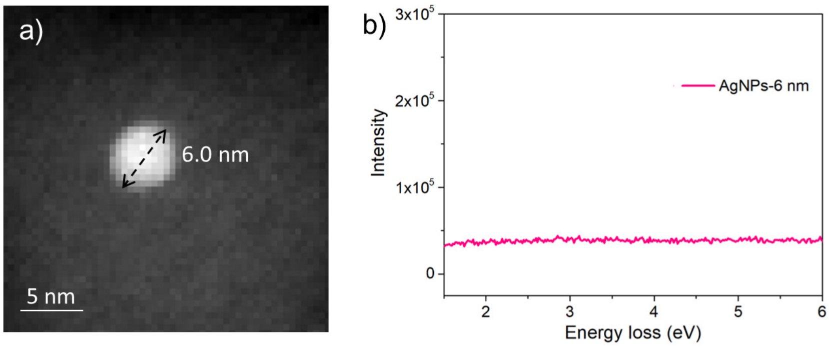

Supplementary Fig. 58 (a) HAADF-STEM image of a representative small AgNP (6 nm) and (b) corresponding low-loss EELS spectrum after background subtraction.

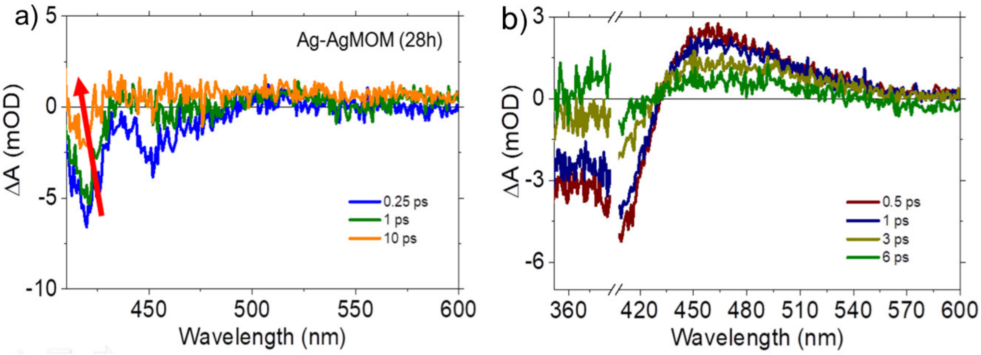

Supplementary Fig. 59. (a) Femtosecond transient absorption spectra at selected time delays of the Ag-AgMOM (28h) sample, taken with a pump wavelength of 400 nm. (b) Femtosecond transient

absorption spectra at selected time delays (0.5, 1, 3 and 6 ps) of AgNPs (strong 400 nm pump scattering data in the range of 389-407 nm was removed for better representation).

We infer that the strong overlap between the negative and positive A signals (in the ranges of ca. 415-424 nm and ca. 430-440 nm, respectively) caused the blue shifts, with respect to the stead-state absorption peak, along with the probe delay time. The small and positive induced absorption signal between 430-440 nm may be hidden under the strong bleaching features centered at 415 nm and 458 nm at an early time delay (<1 ps). As both the bleaching recovers partially at the 10 ps time scale, the appearance of the absorption signal between 430-440 nm becomes clear. It means that the absorption signal between 430-440 nm should also be present at <1 ps, but we cannot resolve it due to strongly overlapping signals.

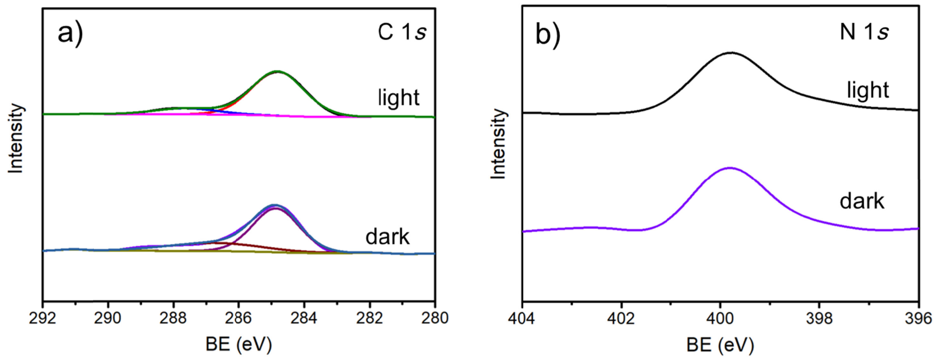

Supplementary Fig. 60 High-resolution XPS spectra for (a) C 1*s* and (b) N 1*s* of Ag-AgMOM (4h) with or without 420 nm LED irradiation (BE: binding energy).

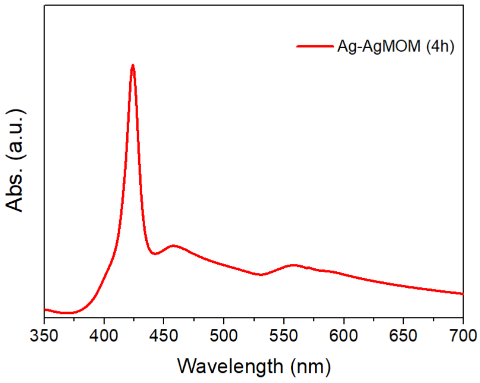

Supplementary Fig. 61 UV-Vis absorption spectrum of Ag-AgMOM (4h) in ethanol solution.

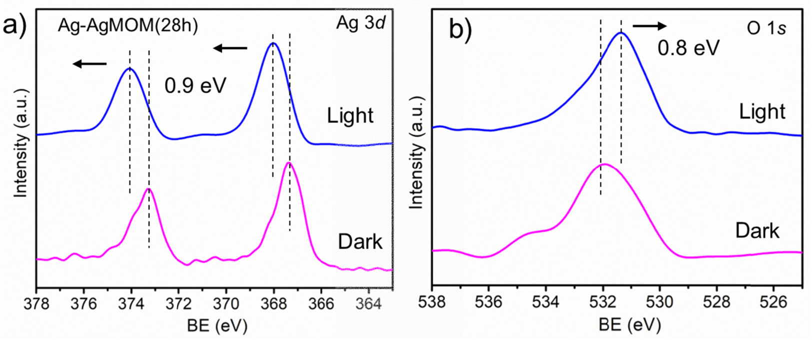

Supplementary Fig. 62 High-resolution XPS spectra for (a) Ag 3*d* and (b) O 1*s* of Ag-AgMOM (28h) with or without 420 nm LED irradiation.

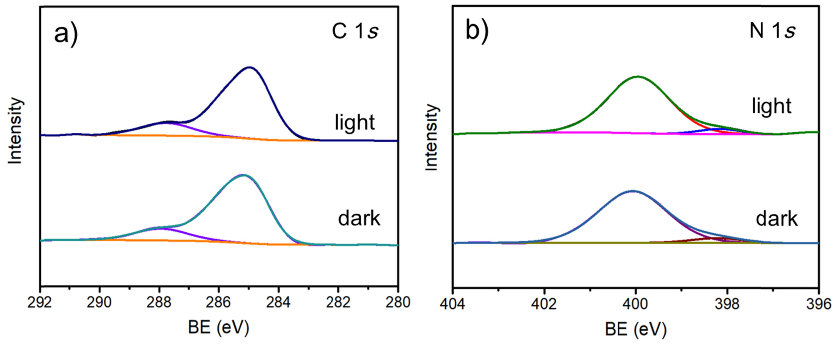

Supplementary Fig. 63 High-resolution XPS spectra for (a) C 1*s* and (b) N 1*s* of Ag-AgMOM (28h) with or without 420 nm LED irradiation.

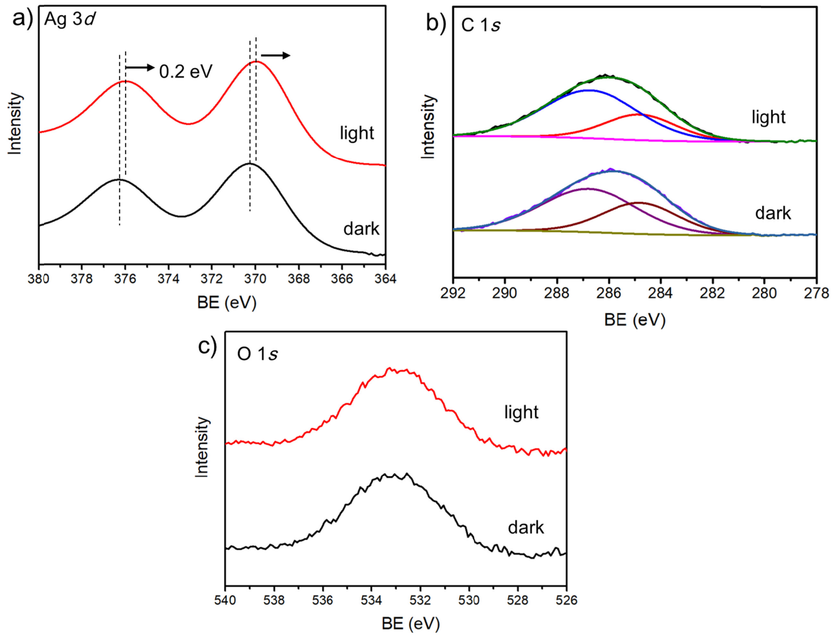

Supplementary Fig. 64 High-resolution XPS spectra for (a) Ag 3*d*, (b) C 1*s* and (c) O 1*s* of AgNPs with or without 420 nm LED irradiation.

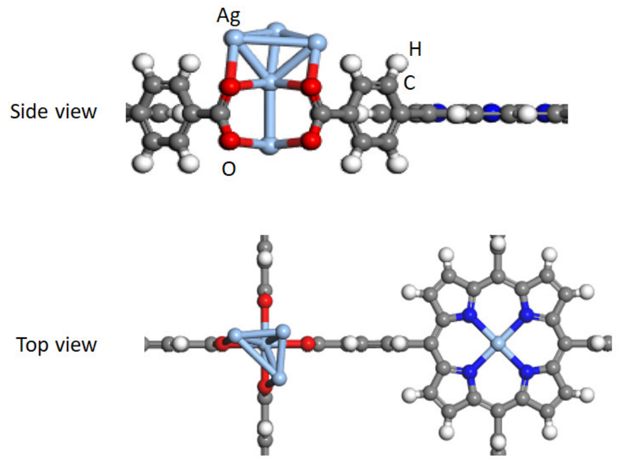

Supplementary Fig. 65 Top and side view of the Ag_3-MOM model.

Supplementary Table 1 CBM and VBM positions of CuMOM and Ag-AgMOM.

	**Band gap**	**CBM (*vs.* NHE)**	**VBM (*vs.* NHE)**
**CuMOM**	1.87 eV	-0.46 eV	1.43 eV
**Ag-AgMOM**	1.59 eV	-1.01 eV	0.58 eV

Supplementary Table 2 Metal-organic material (including MOFs) - based photocatalytic hydrogen generation.

**Photocatalyst**	**H_2 generation rate (μmol g^{-1} h^{-1})**	**Sacrificial reagent**	**Solvent**	**Reference No.**
**Pt@MIL-125/Au**	1743.0 (380-800 nm)	TEOA	18 mL CH_3CN + 0.3 mL water	4
**NH_2 -UiO-66/Pt**	2230 (370-800 nm)	No mention	H_2O/methanol 3:1	5
**Pt@UiO-66-NH_2**	257.38	TEOA	18 mL CH_3CN + 0.2 mL H_2O	6
**Co_2@MIL-125-NH_2**	553	TEA	27.6 mL CH_3CN	7

			+ 1 mL H_2O	
**UCNPs-Pt@MOM/Au**	280	TEOA	18 mL CH_3CN + 0.2 mL H_2O	8
**Al-TCPP-0.1Pt**	129	TEOA	18 mL CH_3CN + 1 mL H_2O	9
**Pt/PCN-777**	586	benzylamine	5 mL DMF+50 L H_2O	10
**HNTM-Ir/Pt**	201.9	TEOA	45 mL CH_3CN +1 mL H_2O	11
**CdS@ZAVCl-MOM**	420	ethanol	9.25 mL H_2O	12
**Ni_4P_2@MOM**	4400(≥ 400 nm)	methanol	H_2O (pH 1.2)	13
**{[Cu^I $Cu^{II}{}_2$ -$(DCTP)_2$] MOM**	32	methanol	90 mL H_2O	14
**Al-TCPP(Zn)/Pt**	200	MeOH	H_2O	15
**Fe_2 (μ-dcbdt)($CO)_6$ /Ru $(bpy)_3$**	280	No mention	1 M acetate buffer (pH 5)	16
**Ni_2P@UiO-66-NH_2**	409.1	TEA	20 mL CH_3CN + 0.2 mL deionized water	17
**$Mo_3S_{13}{}^{2-}$/MIL-125-NH_2**	2094 (≥420 nm)	TEA	CH_3CN:H_2O (79.0:4.9 v/v)	18
**Ni_2P/MIL-125-NH_2**	1230 (≥420 nm)	TEA	0.8 mL H_2O + 13.4 mL CH_3CN	19
**Pt1/SnO_2/UiO-66-NH_2**	2167 (>380 nm)	TEA	4.5 mL CH_3CN + 100 μL H_2O	20
**Al/Zn-PMOF**	200	EDTA	methyl viologen	21

			solution	
**Ag-AgMOM**	1025 (≥ 420 nm)	TEOA	H_2O	This work
**Ag-AgMOM**	3153 (≥ 360 nm)	TEOA	H_2O	This work

Supplementary Table 3 Ag 3$d_{3/2}$ and 3$d_{5/2}$XPS peak positions and peak area ratio of Ag^0-to-Ag ion in Ag-AgMOM (1h) and Ag-AgMOM (20h), which were obtained at the reaction time of 1h and 20h, respectively.

	**Ag^0 3$d_{3/2}$ (eV)**	**Ag^0 3$d_{5/2}$ (eV)**	**Ag ion 3$d_{3/2}$ (eV)**	**Ag ion 3$d_{5/2}$ (eV)**	**Ag^0/Ag ion**
**Ag-AgMOM (1h)**	374.2	368.2	373.8	367.8	27/73
**Ag-AgMOM (20h)**	374.2	368.2	373.6	367.6	68/32

Supplementary Table 4 Extended X-ray absorption fine structure (EXAFS) fitting results.

	**NO (4)***	**RO (Å) (1.997 Å)***	**NAg (12)***	**Rag (Å) (2.851 Å)***	**σ^2 ($Å^2$)**	**E**
**Ag-AgMOM**	0.8 ± 0.2	2.13 ± 0.02	4.1 ± 0.4	2.857 ± 0.007	0.01 ± 0.001	-1.2 ± 0.8

Fitted value for S_0^2 = 0.85. * reference numbers expected for AgO and Ag^0. (NO: the coordination number of O; RO: the distance of O and adjacent atoms; NAg: the coordination number of Ag; Rag: the distance of O and adjacent atoms; Debye-Waller factor (σ^2) and photoelectron energy origin (E)).

4. Supplementary References.

[1] Jiang, Y. *et al.* Simulating Powder X-ray Diffraction Patterns of Two-Dimensional Materials. *Inorg. Chem.* **57**, 15123-15132 (2018).

[2] Wang, Z.-H., Wang, D.-F., Zhang, T., Huang, R.-B. & Zheng, L.-S. Synthesis, Characterization, Crystal Structures and Thermal and Photoluminescence Studies of Dimethylpyrazine-carboxylate Mixed Ligand Silver(i) Coordination Polymers with Various Multinuclear Silver Units. *CrystEngComm* **16,** 5028-5039 (2014).

[3] Sun, D., Zhang, N., Huang, R.-B. & Zheng, L.-S. Series of Ag(I) Coordination Complexes Derived from Aminopyrimidyl Ligands and Dicarboxylates: Syntheses, Crystal Structures, and Properties. *Cryst. Growth Des.* **10**, 3699-3709 (2010).

[4] Xiao, J.-D., Han, L., Luo, J., Yu, S.-H. & Jiang, H.-L. Integration of Plasmonic Effects and Schottky Junctions into Metal-Organic Framework Composites: Steering Charge Flow for Enhanced Visible-Light Photocatalysis. *Angew. Chem. Int. Ed.* **57**, 1103-1107 (2018).

[5] Silva, C. G., Luz, I., LlabrésiXamena, F. X., Corma, A. & García, H. Water Stable Zr-Benzenedicarboxylate Metal-Organic Frameworks as Photocatalysts for Hydrogen Generation. *Chem. Eur.* **16**, 11133-11138 (2010).

[6] Xiao, J.-D. *et al.* Boosting Photocatalytic Hydrogen Production of a Metal-Organic Framework Decorated with Platinum Nanoparticles: The Platinum Location Matters. *Angew. Chem. Int. Ed.* **55**, 9389-9393 (2016).

[7] Li, Z., Xiao, J.-D. & Jiang, H.-L. Encapsulating a Co(II) Molecular Photocatalyst in Metal-Organic Framework for Visible-Light-Driven H2 Production: Boosting Catalytic Efficiency via Spatial Charge Separation. *ACS Catal.* **6**, 5359-5365 (2016).

[8] Li, D., Yu, S.-H. & Jiang, H.-L. From UV to Near-Infrared Light-Responsive Metal-Organic Framework Composites: Plasmon and Upconversion Enhanced Photocatalysis. *Adv. Mater.* **30**, 1707377 (2018).

[9] Fang, X. *et al.* Single Pt Atoms Confined into a Metal–Organic Framework for Efficient Photocatalysis. *Adv. Mater.* **30**, 1705112 (2018).

[10] Liu, H., Xu, C., Li, D. & Jiang, H.-L. Photocatalytic Hydrogen Production Coupled with Selective Benzylamine Oxidation over MOF Composites. *Angew. Chem. Int. Ed.* **57**, 5379-5383 (2018).

[11] He, T. *et al.* Zirconium-Porphyrin-Based Metal-Organic Framework Hollow Nanotubes for Immobilization of Noble-Metal Single Atoms. *Angew. Chem. Int. Ed.* **57**, 3493-3498 (2018).

[12] Saha, S., Das, G., Thote, J. & Banerjee, R. Photocatalytic Metal-Organic Framework from CdS Quantum Dot Incubated Luminescent Metallohydrogel. *J. Am. Chem. Soc.* **136**, 14845-14851 (2014).

[13] Kong, X.-J., Lin, Z., Zhang, Z.-M., Zhang, T. & Lin, W. Hierarchical Integration of Photosensitizing Metal–Organic Frameworks and Nickel-Containing Polyoxometalates for Efficient Visible-Light-Driven Hydrogen Evolution. *Angew. Chem. Int. Ed*. **55**, 6411-6416 (2016).

[14] Wu, Z.-L. *et al.* A Semi-Conductive Copper–Organic Framework with Two Types of Photocatalytic Activity. *Angew. Chem. Int. Ed.* **55**, 4938-4942 (2016).

[15] Fateeva, A. *et al.* A Water-Stable Porphyrin-Based Metal–Organic Framework Active for Visible-Light Photocatalysis. *Angew. Chem. Int. Ed.* **51**, 7440-7444 (2012).

[16] Pullen, S., Fei, H., Orthaber, A., Cohen, S. M. & Ott, S. Enhanced Photochemical Hydrogen Production by a Molecular Diiron Catalyst Incorporated into a Metal–Organic Framework. *J. Am. Chem. Soc.* **135**, 16997-17003 (2013).

[17] Sun, K. *et al.* Incorporating Transition-Metal Phosphides Into Metal-Organic Frameworks for Enhanced Photocatalysis. *Angew. Chem. Int. Ed.* **59**, 22749-22755 (2020).

[18] Nguyen, T. N. *et al.* Photocatalytic Hydrogen Generation from a Visible-Light-Responsive Metal-Organic Framework System: Stability versus Activity of Molybdenum Sulfide Cocatalysts. *ACS Appl. Mater. Interfaces* **10,** 30035-30039 (2018).

[19] Kampouri, S. *et al.* Concurrent Photocatalytic Hydrogen Generation and Dye Degradation Using MIL-125-NH_2 under Visible Light Irradiation. *Adv. Funct. Mater.* **28,** 1806368 (2018).

[20] Sui, J. *et al.* A General Strategy to Immobilize Single-Atom Catalysts in Metal-Organic Frameworks for Enhanced Photocatalysis. *Adv. Mater.* **34,** e2109203 (2022).

[21] Fateeva, A. *et al.* A Water-stable Porphyrin-based Metal-organic Framework Active for Visible-light Photocatalysis. *Angew. Chem. Int. Ed.* **51**, 7440–7444 (2012).
