# Supplementary material for: Silver nanoparticle enhanced metal-organic matrix with interface-engineering for efficient photocatalytic hydrogen evolution
Source: Nat Commun. 2023 Feb 1;14:541. doi: 10.1038/s41467-023-35981-8 (PMC9892045; doi:10.1038/s41467-023-35981-8)
Supplement: Supplementary file 2 — Reporting Summary [file 41467_2023_35981_MOESM2_ESM.pdf]

nature research

Corresponding author(s): Dongling Ma

Lasing Reporting Summary

Nature Research wishes to improve the reproducibility of the work that we publish. This form is intended for publication with all accepted papers reporting claims of lasing and provides structure for consistency and transparency in reporting. Some list items might not apply to an individual manuscript, but all fields must be completed for clarity.

For further information on Nature Research policies, including our data availability policy, see Authors & Referees.

Experimental design

Please check: are the following details reported in the manuscript?

1. Threshold

   Plots of device output power versus pump power over a wide range of values indicating a clear threshold
   - [ ] Yes
   - [x] No

   The object of our study is not a laser device.

2. Linewidth narrowing

   Plots of spectral power density for the emission at pump powers below, around, and above the lasing threshold, indicating a clear linewidth narrowing at threshold
   - [ ] Yes
   - [x] No

   The object of our study is not a laser device.

   Resolution of the spectrometer used to make spectral measurements
   - [ ] Yes
   - [x] No

   The object of our study is not a laser device.

3. Coherent emission

   Measurements of the coherence and/or polarization of the emission
   - [ ] Yes
   - [x] No

   The object of our study is not a laser device.

4. Beam spatial profile

   Image and/or measurement of the spatial shape and profile of the emission, showing a well-defined beam above threshold
   - [ ] Yes
   - [x] No

   The object of our study is not a laser device.

5. Operating conditions

   Description of the laser and pumping conditions
   Continuous-wave, pulsed, temperature of operation
   - [x] Yes
   - [ ] No

   The object of our study is not a laser device.

   Threshold values provided as density values (e.g. W cm^{-2} or J cm^{-2}) taking into account the area of the device
   - [ ] Yes
   - [x] No

   The object of our study is not a laser device.

6. Alternative explanations

   Reasoning as to why alternative explanations have been ruled out as responsible for the emission characteristics
   e.g. amplified spontaneous, directional scattering; modification of fluorescence spectrum by the cavity
   - [ ] Yes
   - [x] No

   The object of our study is not a laser device.

7. Theoretical analysis

   Theoretical analysis that ensures that the experimental values measured are realistic and reasonable
   e.g. laser threshold, linewidth, cavity gain-loss, efficiency
   - [x] Yes
   - [ ] No

   Method part in manuscript and SI

8. Statistics

   Number of devices fabricated and tested
   - [x] Yes
   - [ ] No

   Method part in manuscript and SI

   Statistical analysis of the device performance and lifetime (time to failure)
   - [x] Yes
   - [ ] No

   Method part in manuscript and SI
